# vSNP: a SNP pipeline for the generation of transparent SNP matrices and phylogenetic trees from whole genome sequencing data sets

**DOI:** 10.1186/s12864-024-10437-5

**Published:** 2024-06-01

**Authors:** Jessica Hicks, Tod Stuber, Kristina Lantz, Mia Torchetti, Suelee Robbe-Austerman

**Affiliations:** National Veterinary Services Laboratories (NVSL), USDA, 1920 Dayton Avenue, Ames, IA 50010 USA

**Keywords:** SNPs, Sequencing, Bioinformatics

## Abstract

**Background:**

Several single nucleotide polymorphism (SNP) pipelines exist, each offering its own advantages. Among them and described here is vSNP that has been developed over the past decade and is specifically tailored to meet the needs of diagnostic laboratories. Laboratories that aim to provide rapid whole genome sequencing results during outbreak investigations face unique challenges. vSNP addresses these challenges by enabling users to verify and validate sequence accuracy with ease- having utility across various pathogens, being fully auditable, and presenting results that are easy to interpret and can be comprehended by individuals with diverse backgrounds.

**Results:**

vSNP has proven effective for real-time phylogenetic analysis of disease outbreaks and eradication efforts, including bovine tuberculosis, brucellosis, virulent Newcastle disease, SARS-CoV-2, African swine fever, and highly pathogenic avian influenza. The pipeline produces easy-to-read SNP matrices, sorted for convenience, as well as corresponding phylogenetic trees, making the output easily understandable. Essential data for verifying SNPs is included in the output, and the process has been divided into two steps for ease of use and faster processing times. vSNP requires minimal computational resources to run and can be run in a wide range of environments. Several utilities have been developed to make analysis more accessible for subject matter experts who may not have computational expertise.

**Conclusion:**

The vSNP pipeline integrates seamlessly into a diagnostic workflow and meets the criteria for quality control accreditation programs, such as 17025 by the International Organization for Standardization. Its versatility and robustness make it suitable for use with a diverse range of organisms, providing detailed, reproducible, and transparent results, making it a valuable tool in various applications, including phylogenetic analysis performed in real time.

**Supplementary Information:**

The online version contains supplementary material available at 10.1186/s12864-024-10437-5.

## Introduction

The utilization of genomic data for characterizing microbes in diagnostic and research labs is growing due to the increasing accessibility and declining costs of whole genome sequencing. The analysis of sequences allows for a deeper understanding of complex genetic relationships and, in disease outbreak-associated isolates, can assist in disease tracing and early outbreak recognition. The unparalleled discriminatory power of analysis at the Single Nucleotide Polymorphism (SNP) level makes it a crucial resource in epidemiology [1, 2].

However, the generation of comprehensible and transparent results in an analysis pipeline poses certain challenges such as repeatability, reproducibility, precision, accuracy and manageability. While it is beyond the scope of this paper to review the many tools and pipelines developed and available for SNP analysis, an example of the precision and accuracy of the general method was highlighted in a paper comparing four independently developed SNP analysis pipelines and cgMLST for tuberculosis that found a high degree of consensus in clustering isolates, but more discrimination using the SNP pipelines within clusters[3]. The authors discuss the lack of standardization particularly pertaining to thresholds, depth of coverage, and mixed calls. Consequently, the novelty of an analysis pipeline is not in the analysis itself a but in the processes that allow for documentation (for compete reproducibility as required for accreditation), decision points associated with thresholds and SNP calls, management of the database (reference data set; inclusion/exclusion of isolates), decisions regarding unique areas of the pathogen’s genome (repeat regions, insertions, recombination, etc..) and transparency and readability of the results and output. Additionally, for use in a diagnostic laboratory, a pipeline must be robust and flexible enough to accommodate the wide range of organisms and genetic variability encountered in such a setting while maintaining efficiency in order to respond to rapidly evolving events and impact field decisions.

Different approaches have been employed to manage these challenges. While some pipelines simplify the SNP analysis process by combining all steps into a single step for ease of use, this approach can sacrifice speed and efficiency, particularly with large datasets and when a comprehensive high-resolution analysis is required [4, 5]. Achieving both reproducible and comprehensible output to inform various stakeholders rapidly enough to impact actions in the field is necessary for whole genome sequencing to fully realize its potential within a diagnostic laboratory workflow.

Here, we present a SNP analysis pipeline, vSNP, which meets all of these criteria. To the best of our knowledge, vSNP is the first SNP pipeline that begins with raw sequencing data and at the conclusion presents SNP matrices and accompanying data in an easily reviewable and correlatable format with phylogenetic trees, making the data transparent and readily reportable.

The National Veterinary Services Laboratories (NVSL) initiated development of the vSNP pipeline in 2011. Throughout the ensuing 13 years, various models and programs were evaluated within the pipeline including SNP calling models and phylogenetic tree models. The NVSL was very fortunate to have decades worth of historical isolates for important nationally controlled diseases such as bovine tuberculosis, brucellosis, and contagious equine metritis [6–8]. Along with those isolates, detailed investigation records including animal movement permits, site maps, animal sale records and interviews were available. The isolates along with epidemiological records offered unprecedented data to validate SNP calls and phylogenetic trees using animal transmission events, animals within herds, and multiple isolates from the same animal. It was within this framework that the NVSL accredited the pipeline in 2017 to ISO 17025 standards. Beyond the work of the NVSL, the precision and accuracy of the vSNP pipeline was demonstrated and compared against other popular SNP pipelines commonly used with tuberculosis isolates [9].

## Methods

### vSNP overview

The vSNP pipeline is written in Python 3 and is available from the Anaconda package manager or GitHub (https://github.com/USDA-VS/vSNP3) [10]. It is a package of custom Python scripts that can be run with relatively minimal computational resources (4 cores and 8 GB of memory) but can also be scaled to make use of additional resources, when available, by using multithreading via python packages. vSNP was tested on macOS and Linux distributions, including Ubuntu, CentOS 6 and 7, and Windows Linux Subsytem. Additional GitHub repositories with test data are listed on the vSNP3 GitHub site.

#### Workflow

**Fig. 1:**
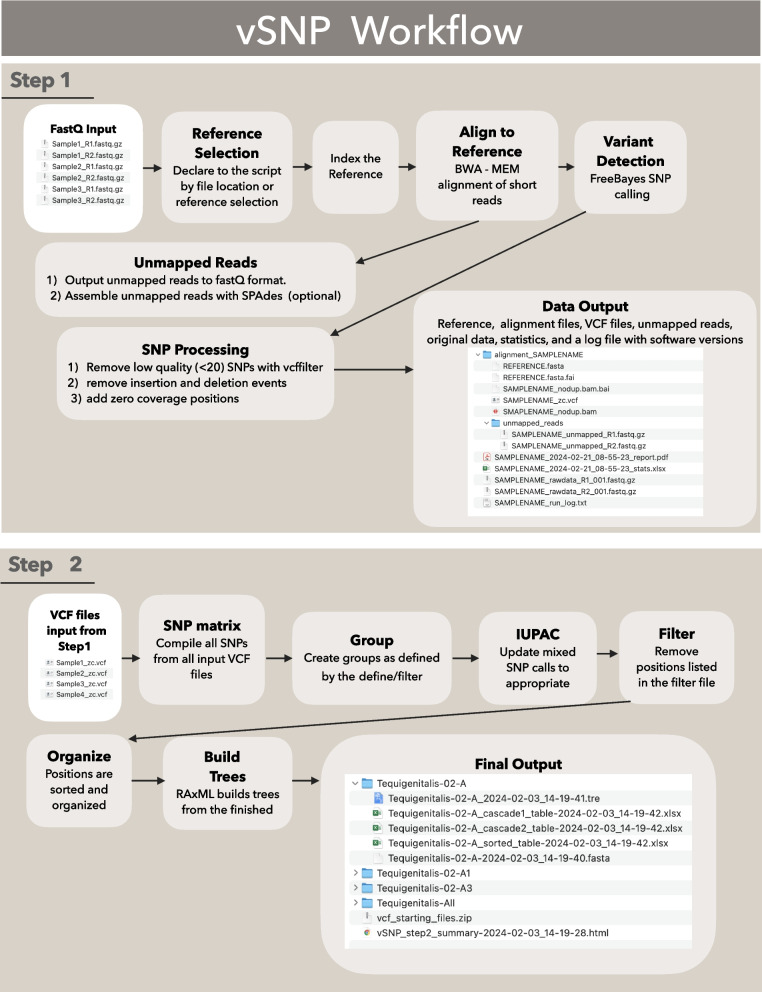
vSNP Workflow Step 1 and step 2 vSNP workflow

The vSNP pipeline divides the process into two steps. Step 1 evaluates quality, aligns raw data to the reference of choice, provides limited characterization, as well as generates variant files in the variant call formant (VCF) for use in Step 2. The Step 1 output enables users within the laboratory to direct and target the comparison of the new VCF files to a specific validated database of VCF files, which occurs in Step 2. (See Fig. 1).

### Step 1

Step 1 initially requires a high-quality reference in FASTA format, which can either be provided by the user or be provided in the dependency files. FastQ files, whether they are paired-end or single-read files, are inputted and separated into individual directories for independent processing. First, the reference is indexed using both Samtools and Burrows-Wheeler Aligner (BWA), followed by the alignment of raw reads using the BWA-MEM algorithm specifically designed for short-read technologies like Illumina (Illumina, Inc., San Diego, CA) and Ion (Thermo Fisher Scientific, Waltham, MA) [11–13]. The next step is the detection of variants using FreeBayes, and low-quality filtering using vcffilter [14, 15]. Sample specific VCF files are produced iteratively for transparency, initially removing SNPs with a SNP Quality (QUAL) score less than 20. In the second iteration, positions with no coverage are added the sample’s VCF file in order to differentiate positions with no coverage from SNP positions and positions that match the reference, these VCFs are referred to as “Zero coverage VCF files”. To ensure completeness, unmapped reads are written to new FastQ files with the option to assemble these reads using SPAdes for convenience, if further investigation of the unmapped reads is desired [16]. At the conclusion of the run vSNP outputs several metrics for evaluating sequencing and alignment and are listed in Table 1. These can be used to evaluate the reference’s suitability for the sample alignment, but the final decision is left to the user since no standard metrics exist that apply to all possible uses for this data. If the reference is deemed unsuitable, the sample can then be aligned to alternate references or the analysis may stop before adding unnecessarily noisy data to downstream analysis. Obviously, the output of Step 1 is specific to the reference alignment. If a sample is aligned to more than one reference, Step 1 must be repeated for each reference, thus generating separate directories. In addition to the raw data and aforementioned statistics, the sample directory includes the reference FASTA, alignment in BAM format, necessary index files, VCF file, and unmapped read data. The directory also contains dependency and software version documentation. Keeping these data in the sample folder allows for easy review and traceability of SNPs and how they were identified as this includes all relevant files for the visualization of alignments and assessment of SNPs or other polymorphisms using tools such as the Integrated Genomics Viewer (IGV) [17]. Zero coverage VCF files for individual samples are then compiled from Step 1 where the same reference was used, and are processed together in Step2.

**Table 1:** Sequencing and Alignment Metrics. The metrics are output in each sample directory at the conclusion of Step 1

Sequncing Metrics	Alignment Metrics
File size(s) of raw data	Mapped Paired Reads
Read count(s)	Mapped Single Reads
Read length sum(s)	Unmapped Reads
Minimum Read Length	Unmapped % of Reads
Average Read Length	Unmapped Assembled Contigs
Maximum Read Length	Duplicate Pair Reads
% Passing Q20	Duplicate Single Reads
% Passing Q30	Duplicate % of Mapped Reads
Average Read Quality Score	Reference Length
	% of Reference with Coverage
	Average Depth of Coverage
	No Coverage Bases
	% of Reference with No Coverage

### Step 2

Figure 1 also outlines Step 2 of the vSNP pipeline, which computes aligned FASTA files, SNP matrices, and phylogenetic trees from a group of Zero coverage VCF files output from Step 1. All sample specific VCF files to be analyzed together must be called from alignments to the same reference. SNPs, including those with mixed calls, represented by an allele count (AC) of 1 are identified. If a SNP call has an AC = 1 at a position that is parsimony informative in the group, the allele nucleotide call is changed in the SNP matrices to an ambiguity code representing the mixed SNP as referenced in the International Union of Pure and Applied Chemistry (IUPAC) nucleotide ambiguity codes, leaving the original VCF files unchanged. SNP matrices are then used to build phylogenetic trees with RAxML using the GTR-CATI model of substitution. In addition to these SNP matrices and phylogenetic trees, Step 2 documents software dependency versions, creates a summary of the samples processed as well as a zip archive of the VCF files used in the analysis to ensure traceability, and creates a static copy of data used in each analysis.

The multiple outputs of Step 2 are intended to improve utility of the data. SNP matrices are presented with three different sorts. The first SNP matrix is organized in a so-called “cascading” format, with the most common SNPs appearing first in the matrix, followed by less common and unique SNPs to each sample. This format enables easier visualization and comparisons of SNPs amongst the input group of isolates. Evolutionary SNP patterns are visually evident when organized in this manner, making it apparent when a SNP or a set of SNPs are multiphyletic, suggesting selection pressure. The second matrix is organized in a similar cascading format, but with a different approach in an attempt to identify poor samples. The final matrix is sorted by reference position, making it easy to inspect regions of the reference genome across isolates. All SNP matrices include reference positions, map quality scores, and annotations, where available, to allow for quick evaluation of SNPs based on the location relative to the genes identified.

The script for Step 2 is initiated independently of the Step 1 script. Since vSNP uses sample specific VCF files, the sample set included in the Step 2 analysis can easily be modified to include new samples or subsets of samples. Independence of Step 2 also allows a user to examine Step 2 output and add filters or define groups with the utilities described below, repeating only Step 2 for these to be applied. By not repeating Step 1, alignment of the raw data is avoided, increasing the efficiency of the pipeline.

### Utilities

The vSNP pipeline provides several utilities to expand on the described base functionality and increase ease of use as well as flexibility. Firstly, to ensure references are easily accessible and reference dependent files can be associated with the appropriate reference (including the spreadsheet utilities described below), vSNP provides a utility to use reference directories. Stored in commonly accessible locations, these directories are defined for each reference and contain the reference FASTA, a GenBank formatted file with annotations, and spreadsheets that run additional utilities. This avoids copying reference information for each run and reduces maintenance of data to a single directory for each reference. If reference directory framework is used, the reference name can be declared as an input option at either step, prompting the script to use all available files in the reference directory. If the reference file setup described is not used, a reference FASTA file and location must be specified as an input to Step 1, and a GenBank formatted file may be used as an input option to Step 2.

The spreadsheet-based utilities provide impactful control parameters in a straight-forward manner allowing for ease of use by genomics or organism subject matter experts that are non-computational. Firstly, problematic SNPs or regions such as mobile elements identified through manual review can be filtered from future analyses using a spreadsheet denoting the reference position or range of positions to be excluded. In this same spreadsheet, groups may also be defined by designating a common SNP location and a name applied to the group for output clarity. The creation of these groups produces smaller, more manageable SNP matrices and trees of genetically related isolates containing the defining SNP during Step 2. Filters may be applied to groups independently to provide flexibility in evaluating differences between groups. While the grouping utility primarily enhances the functionality of Step 2, it is also used in future analysis of samples through Step 1 with the inclusion of a list of qualified groups for each sample in the statistics file. If no groups are defined for a reference, then the only group will include all samples that were placed in the analysis; and if no defining SNP exists for a sample, it will only be included in the group with all samples.

Additionally, removal of isolates from all future analyses can be performed from a separate spreadsheet designated for this purpose. This ensures any files that have known problems will not accidentally end up in future analyses. Finally, a spreadsheet may also be employed to update sample names with metadata tags during processing, avoiding time-consuming manual updates to file names. Collectively, the spreadsheets to run these utilities along with the reference in FASTA format and the gbk formatted annotation file, when available, are referred to as the dependency files.

Additional utilities can be declared when scripts are initiated, especially to enhance flexibility of Step 2. For example, the option to run Step 2 without the manually defined list of filters creates an system of removing filters on a single run basis without the need to delete or remove the spreadsheet. Also, the quality threshold at which a mixed SNP is changed to “N”, the any-base designation, can also be adjusted at script initiation for Step 2.

The organization and maintenance of files throughout the pipeline accommodates curation and investigation of SNPs. While initial review of SNPs is likely to be performed in the SNP matrices of Step 2, the data from Step 1 is maintained for further interrogation of positions.

## Data sets

In order to demonstrate workflow, features, and broad applicability of the vSNP pipeline, multiple sets of publicly available test data were compiled (Supplemental Table 1). These sequences represent extremely small and concise groups for ease of explanation and to assess the utility / value of the SNP matrices in the interpretation of the data, particularly with mixed SNPs, as well as to assess the utility of SNP matrices beyond the phylogenetic tree. The first dataset contains 11 *Mycobacterium bovis* samples and are aligned to reference *M. bovis* AF2122/97 (NCBI accession NC_002945.4). The second dataset consists of multiple *Taylorella equigenitalis* samples from three animals, two females that had been bred to a single male and are aligned to NCBI accession NZ_CP021060.1. The third dataset consists of seven SARS CoV-2 samples isolated aligned to NCBI accession NC_045512.2. The final dataset consists of 32 Newcastle disease virus samples aligned to NCBI accession OR881391.

Four publicly available datasets of *Mycobacterium bovis* were used to assess processing times of the vSNP pipeline. All datasets contained Illumina Miseq paired read data (Illumina, Inc. San Diego, CA). One dataset contains 48 samples and an average file size of 214 MB, the second dataset contains 81 samples with an average file size of 259 MB, the third set contains 136 with an average file size of 123 MB and was previously publish using this pipeline [18]. The final data set contains 287 samples with an average file size of 239 MB and was also previously published [19]. Additional information is available in Supplementary Table 2.

## Results

The following results are the output of Step 2, which is considered the final output of the pipeline and is the point at which interpretation occurs. These data represent specific cases that have been related together through use of the grouping function.

### *Mycobacteriumbovis*

The dataset presented in Table 2 contains samples from three human patients associated with an outbreak in Nebraska that involved human-to-human transmission [20] and several dairy cattle originating from an endemic dairy farm in Nuevo Leon, Mexico. The dairy cattle were not epidemiologically related to the human patients, they just happened to be the most closely related isolates in the USDA’s database and suggests the original spillover event into humans likely came from consuming dairy products from Mexico. These cattle sequences also demonstrate the exposure humans have when consuming unpasteurized milk from larger dairy farms infected with *M. bovis*, as multiple strains are likely contained within a single dairy product. As detailed in the referenced paper, Patient A was thought to infect Patient B, who then in turn had contact with Patient C. Unfortunately, both Patient A and Patient C isolates were sub-cultured from liquid media on to Lowenstein-Jenson (LJ) media prior to sequencing. This LJ media allowed for single colony selection, potentially misrepresenting the diversity in the clinical samples. Patient B’s sample was sequenced directly from the primary liquid media, preventing this laboratory bias from occurring. Consequently, mixed SNPs that were present in Patient B were identified as consensus SNPs in Patient C, supporting the directional transmission from Patient B to Patient C. Unfortunately, the laboratory methods may have impacted the inability to link Patient A to Patient B. *Mycobacterium bovis* is not known to readily transmit from person-to-person; the significant accumulation of SNPs between patients may represent a lack of evolutionary fitness or may represent the diversity of the population within Patient A. The cattle isolates also show active transmission and mixed strain infections. These mixed SNP positions are unable to be depicted in the phylogenetic tree, forcing a loss of information important in understanding the relationships between these isolates. For this reason, the SNP matrix provides a more definitive view of the relationships.

**Table 2:**
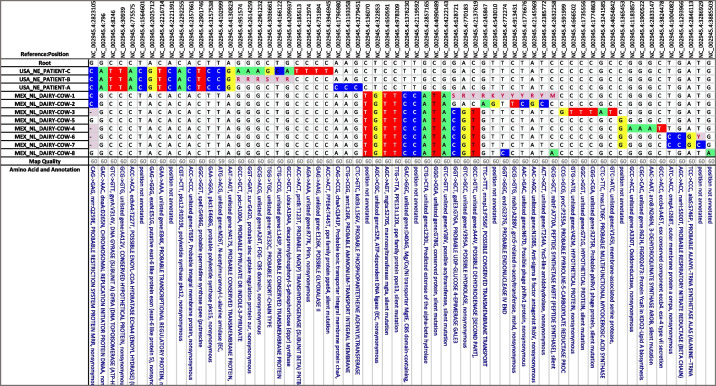
SNP matrix of *M. bovis *from an outbreak involving 3 human patients and 8 dairy cattle. Although these isolates have no epidemiological link, the genetic relatedness is evident in this matrix

### *Taylorella equigenitalis*

**Fig. 2:**
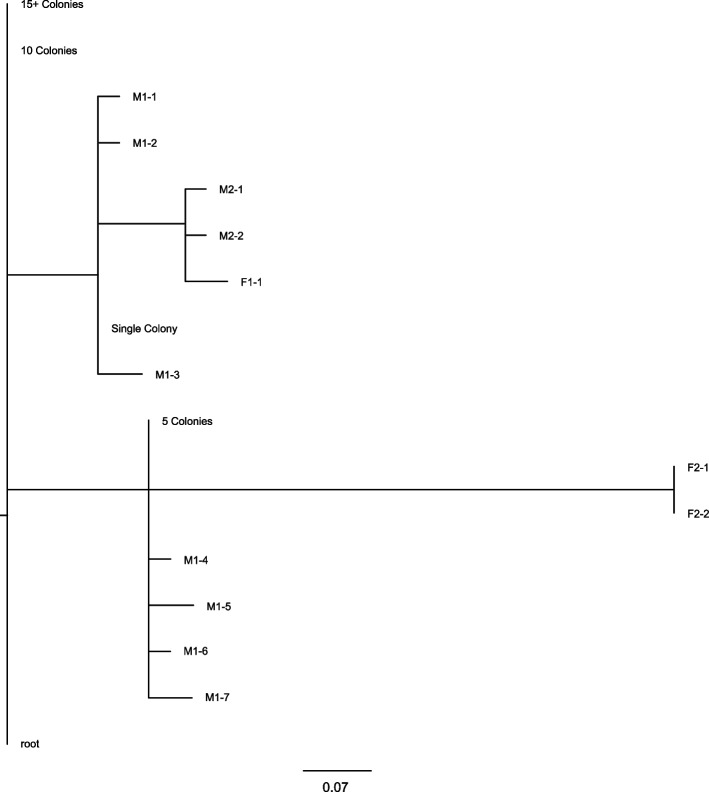
The phylogenetic tree of *T. equigenitalis* based on the SNP table (Table 3)

Inspection of a sequence dataset from *T. equigenitalis* isolates further details the significance of the SNP matrices (Table 3) in clarifying relationships beyond what is possible to visualize with a phylogenetic tree, especially when isolates are closely related (Fig. 2). In this outbreak of *T. equigenitalis*, an epidemiological investigation linked 3 horses to a positive male horse believed to be the source of the outbreak. The initial samples from the male horse and an associated stallion (samples M1-1, M1-2, M2-1, and M2-2), sequenced from purified culture, showed a clear genetic relationship with the one of the female horses (sample F1-1) in both the SNP matrix and phylogenetic tree, but they showed a more distant genetic relationship with samples from the second female (F2-1 and F2-2). However, breeding records indicated the second female had only been bred by the first male. In an attempt to further elucidate the genetic relationship between the samples from the first male and second female, several semen straws were cultured (1 straw per plate); and multiple single-colonies were sequenced from each culture plate. These samples are labeled M1-3, M1-4, M1-5, M1-6, and M1-7 with samples M1-4 thru M1-7 displaying a slightly different genotype from the previous male samples, but this genotype is more closely related to the second female. Additionally, a set of four samples were sequenced based on a single colony, five colonies, ten colonies, and 15 + colonies from a single culture plate to determine if both strains were present in a single straw and how many colony selections would be necessary to capture this diversity in a single sequenced sample. These samples are labeled according to their colony count. Unexpectedly, the single colony sample is very closely related to a group of other single colony samples from that male. However, the “5 Colonies” sample has mixed SNP calls at only 8 of the 10 positions differentiating the 2 strains, while the sample with “10 Colonies” has mixed calls at all 10 positions, 1 of those positions is called “N” due to mixed calls and a low quality score at that position. Finally, the sample with “15 + Colonies” has the correct mixed base call at all 10 positions.

**Table 3:**
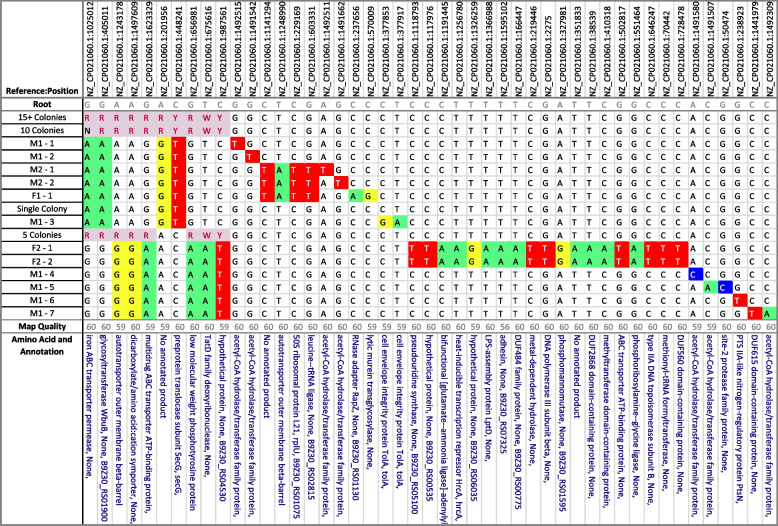
The SNP matrix of *T. equigenitalis* isolates for an outbreak involving 2 stallions and 2 mares. This matrix depicts the diversity of *T. equigenitalis* found in the M1 individual and the transmission to the 2 mares

The phylogenetic tree and SNP matrix generated from this analysis showed diverging but related strains from the single-colony semen samples, each strain relating to a single female. In contrast, the phylogenetic tree displays potentially misleading placement of the multi-colony semen samples that the SNP matrix further resolves. The inability of the phylogenetic tree to display mixed SNP positions causes these mixed isolates to be placed at the parent node, as opposed to the SNP matrix, which clearly shows the mixed status of these isolates among key SNP positions.

### SARS-CoV2

The analysis in Table 4 shows selected SARS-CoV-2 sequences from animal samples. The samples include a subset that are part of the delta variant of concern (Pango lineage B.1.617.2 and AY lineages), as well as a single sample from a non-delta lineage, hCoV-19/cat/USA/VA-20–037760-004/2020, Pango lineage B.1.240.

The difficulties with mixed SNP positions in the previous *Mycobacterium bovis* example do not exist in this viral example. However, it is worth noting that the positions with mixed calls in this matrix also have mixed calls in the isolates where a SNP is listed. For example, position 22,995 has both “A” and “C” calls in the isolates in rows 6, 7, and 8, but the number of “C” calls falls below the threshold used by FreeBayes to call the position mixed. A SNP that is approximately 95% alternate calls is generally called pure, while a SNP with only approximately 90% alternate calls will be called mixed. It can be important to consider this when examining a position with mixed calls.

Samples hCoV-19/lion/Puerto Rico/PR-21–31,013-001/2021 and hCoV-19/lion/Puerto Rico/PR-21–31,013-006/2021 are an example of two samples from animals at the same site. These samples are from a single premises and have zero SNP differences. In addition, they are delta variant, which was the predominant circulating variant at the time. This can easily be confirmed by identifying the characteristic SNPs in the spike protein, namely G142D, L452R, and T478K for the delta variant, either by assigning clades to those defining SNPs, or viewing the annotation on the cascade matrix. This example demonstrates the utility of the SNP-based clade assignment for aligning sequence results to standardized nomenclature structure.

**Table 4:**
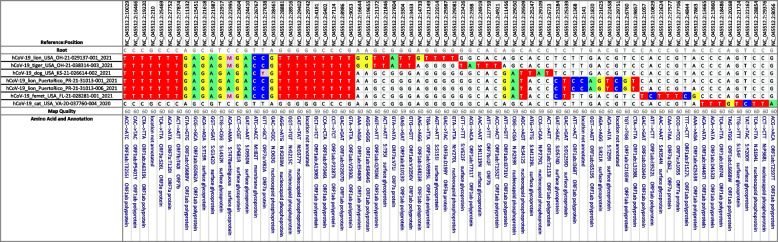
This SNP matrix depicts the relationship between several strains of the SARS-CoV-2 delta variant, as well as their genetic distance (in SNPs) to a non-delta lineage

### Newcastle disease outbreak, California event 2019

**Fig. 3:**
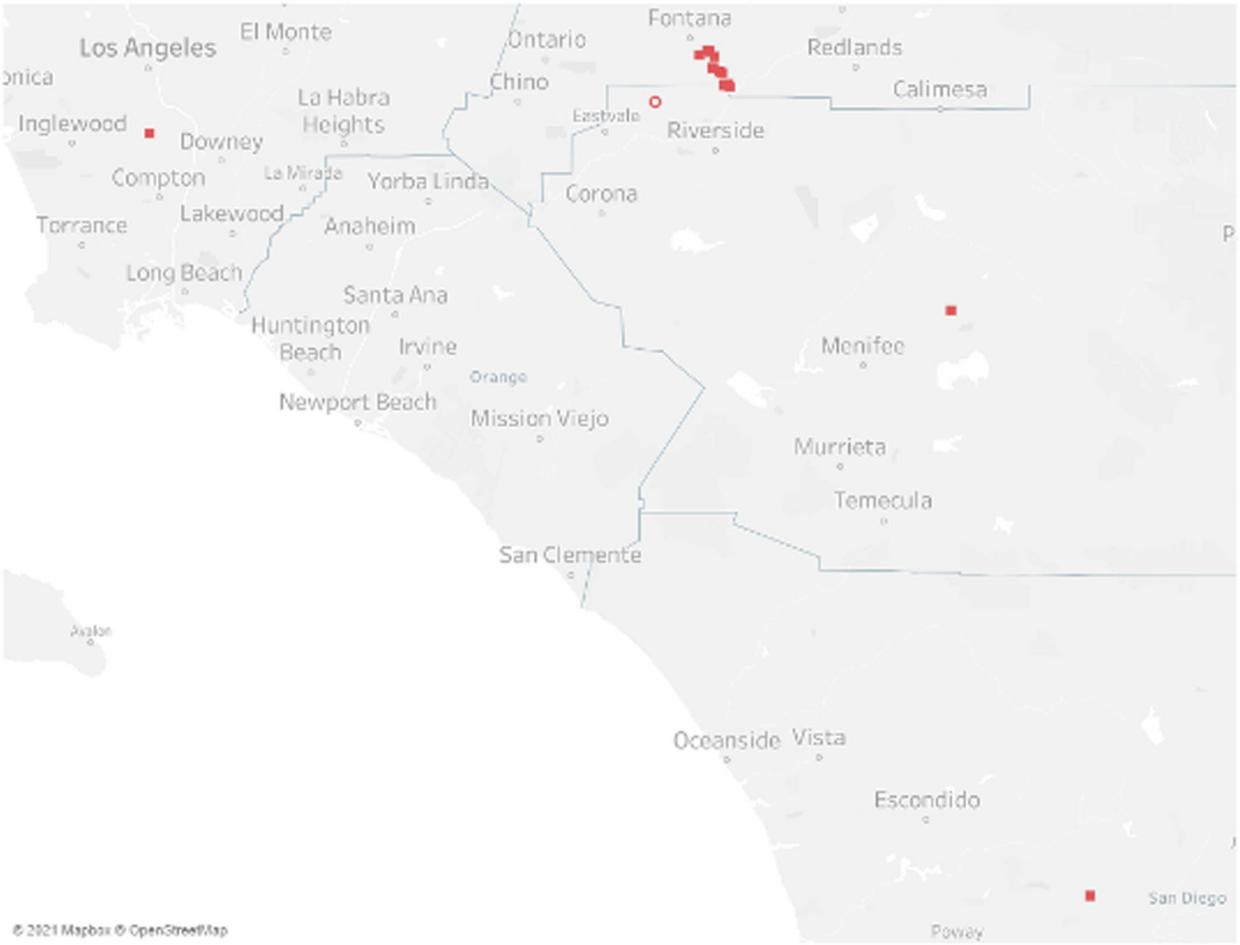
Distribution map of the cluster of highly related vNDV-02 viruses which occurred after August 1, 2019. The open red circle indicates the location of an earlier detection of this virus cluster prior to its resurgence in August

Table 5 and Fig. 3 display a subset of data from the virulent Newcastle disease (vND) outbreak in California during 2019–2020. The CA2018 virus (genotype Vb) is related to older Central American village poultry viruses (Belize 2008, Honduras 2007) and one from the U.S. (smuggled parrot 1996, CA2002), which represent viruses from birds with low or no vaccine coverage [21]. The genetic analysis from the outbreak included 446 full genome sequences and supported a recent, single introduction into California followed by secondary spread.

The vSNP analysis was used in real-time during the outbreak to monitor viruses and to flag potential directional movements of the virus for further epidemiologic investigation. Using vSNP with verification from other phylogenetic tools, it was discovered that the virus diverged early on into two lineages. Over the course of the outbreak, several different sublineages evolved, contributing to genetic diversity overall. However, toward the end of the outbreak, a single cluster of highly similar viruses was detected. Epidemiologic investigations did identify connections among many of these last detections, which were ultimately stamped out.

**Table 5:**
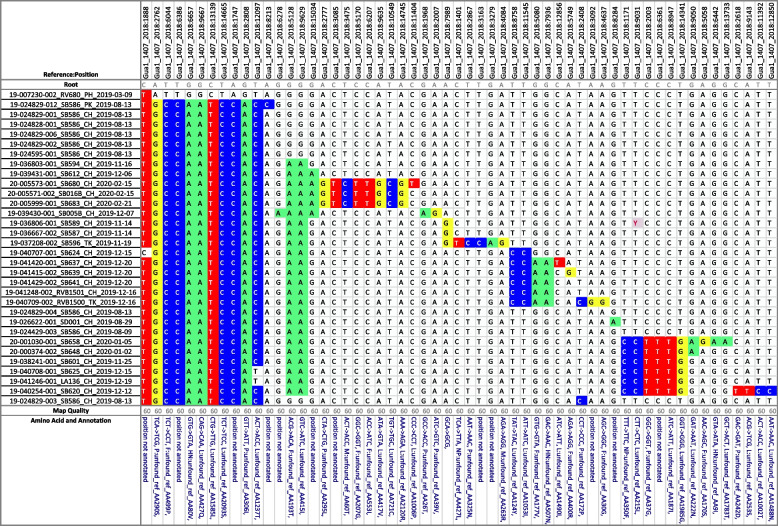
This SNP matrix depicts the relationship between several viruses from the California 2019-2020 Newcastle disease outbreak later in the event when a single cluster of viruses was circulating

## Performance

The four sets of *M. bovis* sequences were successfully executed for runtime comparison. Run times for Step 1, Step 2 with the generation of a SNP matrix containing all samples as well as predefined groups, and Step 2 with only predefined groups (all samples were assigned to at least one group) are listed in Table 6. Number of SNPs contained in the SNP matrix with all samples is also listed for better understanding of size and complexity of the SNP matrices. Samples were run on an HPC with 50 compute nodes (2 × Intel Xeon Silver 4214 CPUs, 768 GB DDR4 memory, and 1 TB SSD scratch storage).

**Table 6:** Data attributes and run times. Data sizes and run times for example datasets used to demonstrate processing times of the vSNP pipeline

BioProject	No. of Samples	Step 1 Runtime	Average Fastq File Size	No. of SNPs	Step 2 Runtime with "All" Matrix and Grouping	Step 2 Runtime with Grouping Only
PRJNA693806	48	4.5 min	214 MB	2,610	37 s	26 s
PRJNA622511	81	6 min	258 MB	1,791	46 s	32 s
PRJNA804719	136	4.5 min	123 MB	1,369	2 min 4 s	1 min 47 s
Multiple, See Reference	287	10.5 min	239 MB	11,956	8 min 4 s	2 min 7 s

## Discussion

The vSNP pipeline was developed over many years, making continual improvements to increase flexibility, incorporate new tools, and improve efficiency. While the core concepts of vSNP have remained constant throughout its evolution, the move to vSNP3 brought new and updated command line options. These options made it possible to specify annotation files from the command line without the need to fully setup the reference directory feature described earlier, this allows for easier impromptu use and less overhead for sporadic cases or evaluation of references for suitability. There are also continual enhancements to operate in more advanced computing environments.

There are several features of vSNP that distinguish it from other pipelines. One of these features is its output. The vSNP pipeline is organized and thorough in its output, providing a detailed look at the data generated in each step. This unique setup creates transparency and allows for a highly discriminatory review of the information, enabling data-driven decisions. The maintenance of files necessary to view alignments in a concise manner from Step 1 simplifies data storage and creates detailed documentation including versioning and maintaining critical files for traceability. The output of SNPs into sample specific VCF files, allows samples to be added and removed from future analyses without repeating sample alignments, the process in Step 1 that is the most costly in time and computational resources. In Step 2, SNP matrices with annotations and position information make it easy to review the data. This works well in a typical diagnostic laboratory workflow where samples are collected at different times, and each analysis needs to be cumulative. SNP matrices sorted using RAxML put the matrix in approximate evolutionary order, which makes it easy to identify common phylogenetic groups, homoplastic SNPs that do not follow evolutionary order, and SNP positions that warrant further investigation. The presence of annotation data for each SNP allows for easier evaluation of irregular or homoplastic SNPs, resulting in an informed decision on filtering those positions. The SNP matrices correlate SNP positions with branches in the phylogenetic tree and make use of additional information such as SNP quality, depth of coverage, and mixed SNP positions that cannot easily be depicted in a phylogenetic tree. This creates transparency in the data and provides tangible information about genetic change at the finest resolution possible making it easier to communicate to stakeholders.

In general, nearly all modern SNP pipelines demonstrate a high degree of accuracy, and vSNP in an independent comparison, performed acceptably and within the scope of other pipelines. [3, 9]. The selection of a pipeline depends on use case. vSNP was specifically designed for the diagnostic laboratory, allowing for additional sequences to be easily added or removed as mentioned above. It also calls SNPs liberally and produces outputs (SNP matrices) that allow the laboratorian to easily identify anomalies and correct errors. In addition to the SNP matrices output in Step 2, vSNP Step 1 data contains the files necessary to view alignment maps for manually review of the SNP positions and genome alignments in detail. Through this process users gain familiarity with the data that can also be helpful in understanding genetic shift and other features in the target genome. This process contributes to the accuracy of the final SNP matrix, which is largely dependent on manual curation of the SNPs by a subject matter expert as minimal filtering is initially performed. For diagnostic use, when analyzing closely related isolates or potentially mixed infections, the ability to evaluate individual positions and assess quality attributes of these positions in the SNP matrix has been more informative than relying on phylogenetics and the application of bootstrapping to assess confidence. vSNP use has been documented in several publications describing disease outbreaks. Sequences for these outbreaks have been made public, and these sequences have been used in many other publications with other pipelines [22–26]. Additionally, the usefulness of vSNP has been demonstrated in many research projects both for genomic epidemiology and to evaluate changes in genetic regions of interest [27–30].

The ability to create phylogenetic groups based on defining SNPs is novel to vSNP. This function allows the user to create groups based on genetic relatedness that correlate with branches of the phylogenetic tree or a common genetic element of interest. Inclusion in a group does not exclude isolates from inclusion in additional groups; and, with the ability to assign names to these groups, this unique function allows data to be automatically grouped and organized in future runs once defined. Use of this function to define all the major branches, especially of well characterized trees, can decrease the need to generate large, convoluted SNP matrices with isolates that are known to be more distantly related. Not only does this simplify the output, it also decreases run time of the analysis. Some examples of when this can prove particularly useful include disease outbreaks, when groups correlate with regions of origin, or when looking for a particular genetic marker. This feature can also be used to “zoom in” on increasingly smaller groups of isolates creating progressively smaller and more focused SNP matrices that contain only positions relevant to differentiating isolates in the smaller group. For example, an initial group may be defined by a SNP on a branch near the root, and additional groups may be defined along branches from subsequent nodes. The smaller SNP matrices created in this manner make explaining data simpler and provide more confidence in the placement of the isolate because it should be contained in each group from the highest level until its final placement. While it is not enforced by the pipeline that an isolate must contain the defining SNP of each group in a hierarchy, it can be managed outside of the bioinformatics workflow by a subject matter expert simply altering dependency tables. For example, this function may be used to create a group to identify isolates with a specific ribosomal mutation that confers antimicrobial resistance.

While the vSNP pipeline is intended to run as described above, it also has broad applicability. For this reason, the output of the steps includes files that make additional analyses possible. In particular, the aligned SNP file is provided in FastA format should a user desire to perform model selection, boostrapping, or consensus tree calling. These outputs may also be used as inputs for further analysis with other software.

### Performance

By dividing the process, vSNP avoids unnecessary recomputing at several steps including alignments and SNP calling. Only new, raw data sequences are processed through Step 1, and sample specific VCF files are accumulated for Step 2 processing. This ability to build on previous analyses makes the addition of samples in time-crucial situations simple without compromising the fundamental clarity of results.

Run-time of the vSNP pipeline is dependent on several factors, such as genome size, sequencing depth, number of SNPs called, overall size of the SNP matrices, and computational resources available for running the pipeline as well as how those resources are managed. For general time comparison, four sample datasets with 48, 81, 136, and 287 *Mycobacterium bovis* samples were processed through vSNP. Samples were grouped into processing jobs with up to 5 samples assigned to a processing node. This assignment scheme lead to similar processing times across all example datasets for Step 1 with the final dataset taking the longest time to completion, likely due to 2 factors: the first being there were more jobs than nodes, so 1 node had to process 2 jobs, and the second factor was the large file sizes of a few samples in that data set (6 samples had paired files approximately or greater than 500 MB).

As for the Step 2 run times, all datasets were processed with relatively little time, but the time savings for processing groups of SNP matrices and avoidance of 1 large matrix containing all samples begins to become apparent on the largest dataset. Since the overall tree topology of *Mycobacterium bovis* is well researched, groups were assigned based on SNPs occurring on all major branches and subsequent clades as relevant to current and previous diagnostic cases and research projects. All isolates indicated they belonged to a defined branch at the conclusion of Step 1, this eliminated the need to generate a single SNP matrices and phylogenetic tree for all samples in the analyses. Instead, data was output based on groups only. This decreased overall run time by almost 74% for the largest dataset in the example when compared to running the same dataset and creating these outputs for all data in the analysis in a single matrix. Obviously, the realization of this time savings is still dependent on many parameters, as noted previously, but this level of time savings allows for the processing of large numbers of samples in an efficient way avoiding the process of selecting subsamples or removing samples as datasets grow.

### vSNP limitations

#### Reference selection

vSNP’s ability to produce high resolution genome analysis comes with some limitations. The pipeline is reference-dependent; and therefore, genomes not closely related to the reference will result in poor alignment and a high number of unreliable SNPs, an undesirable characteristic of a dataset for SNP analysis. In general from our experience, SNP counts greater than approximately 10% of the genome size will require significant manual curation and will likely introduce more unresolvable error. Based on experience with viral genomes, a similar virus, e.g. a virus early in an outbreak, is optimal for efficient use of this pipeline; and the major limitation to utility is when sufficient genetic distance accumulates such that the samples no longer assemble reliably to that reference. Ideally, a reference would sit basal to the isolates being aligned in a phylogenetic tree in order to form a properly rooted tree; however, this is not always available, and an additional outgroup isolate may be needed in order to root the tree.

#### Running the pipeline

Running the vSNP pipeline on the command line requires knowledge of the command line to install the software and process samples. Managing errors will require knowledge of the Python programming language.

#### SNP curation

The ability for manual curation of SNPs is an important feature of the vSNP pipeline. Since this is a time-consuming step, many other SNP-finding pipelines exclude this capability in favor of easily derived outputs. However, as evidenced from the use cases above, careful review of outputs often reveals sequencing and SNP calling errors that may be readily identified and corrected to ensure accuracy in SNP calls and resulting matrices. While manually curating SNPs does cause some discomfort and concern with reproducibility, within vSNP, it is very easy for the user to remove filtered positions and repeat the analysis to determine if over filtering was applied. Furthermore, SNPs that were corrected or not corrected within the VCF files are continuously re-evaluated when closely related organisms are added. Using a manual curation process also allows coding to remain more simplified and transparent as complex decision-making algorithms would likely be necessary to automate curation for the wide variety of organisms for which it is regularly used. However, the manual process requires knowledge of the organism, microbial genetics and/or genomics, and sequence platform biases to make well-informed decisions. The level of effort put towards SNP curation is at the decision of the user, but the required effort to complete this process is often justifiable because of the increased requirement for accuracy of and confidence in the results of the analyses in diagnostic laboratories. The added advantage of sharing filtered and curated SNP results as variant matrices in a relatively easily visualizable format is of particular use when sharing and describing results to non-expert stakeholders.

#### Phylogenetics

Currently, the vSNP pipeline uses the GTR-CATI approximation for all phylogenetic tree building to avoid adding additional time for model selection or building trees with models that may be more computationally intense. In our experience this model has produced accurate trees that are comparable to trees made with evolutionary models derived from model fitting. We are not the first to notice parameter rich models often produce sufficiently good trees [31]. An example of the tree in Fig. 3 generated in RAxML under the JC69, GTR-GAMMA, and GTR-CATI models demonstrates the consistency of the trees regardless of the model on even such a small dataset where the GTR-CATI approximation is most likely to perform poorly (See Supplemental Document 2).

## Future updates

A current option exists for long read alignment with Minimap2, but SNP calling and management has not been optimized for this technology [32]. Comparison of SNPs between short read and long read technologies is not currently advisable with this pipeline. Additional SNP callers may also be useful for deep sequencing and data sets not obtained through purified culture. Further enhancements to vSNP will improve this function for more flexible, reliable analyses.

## Conclusions

The vSNP pipeline provides many unique features and flexibilities that make it an adaptable tool in a diagnostic laboratory. The pipeline’s ability to quickly process data in rapidly evolving situations is an imperative attribute, as is the detailed data output that can be understood to many people with diverse backgrounds. These characteristics also make the pipeline very versatile in situations beyond its initial intended use.

## Availability and requirements

*Project Name*: vSNP.

*Project home page*: https://github.com/USDA-VS/vSNP3

*Operating System(s)*: Windows, Linux, Unix.

*Programming Language*: Python.

*Other Requirements*: None.

*License*: None.

*Any restrictions to use by non-academics*: None.

### Supplementary Information

Supplementary Material 1.

Supplementary Material 2.

Supplementary Material 3.

Supplementary Material 4.

## Data Availability

The sequences used in this paper are available from the NCBI Sequence Read Archive (SRA). The use cases are available from BioProjects PRJNA251692 (*Mycobacterium bovis*), PRJNA436694 (*Taylorella equigenitalis*), PRJNA1023551 (SARS-CoV-2), PRJNA1023835 (Newcastle Disease virus). Data sets used for runtime comparison are also available from the NCBI SRA under BioProjects PRJNA693806, PRJNA622511, PRJNA804719, PRJNA449507, and PRJNA251692. The specific SRA accession numbers associated with each sample are listed in Supplementary Table 1.

## References

[1] Salvador LCM, O'Brien DJ, Cosgrove MK, Stuber TP, Schooley AM, Crispell J (2019). Disease management at the wildlife-livestock interface: Using whole-genome sequencing to study the role of elk in Mycobacterium bovis transmission in Michigan, USA. Mol Ecol.

[2] Allard MW, Luo Y, Strain E, Pettengill J, Timme R, Wang C (2013). On the evolutionary history, population genetics and diversity among isolates of Salmonella Enteritidis PFGE pattern JEGX01.0004. PLoS One.

[3] Jajou R, Kohl TA, Walker T, Norman A, Cirillo DM, Tagliani E (2019). Towards standardisation: comparison of five whole genome sequencing (WGS) analysis pipelines for detection of epidemiologically linked tuberculosis cases. Euro Surveill.

[4] Davis S, Pettengill JB, Luo Y, Payne J, Shpuntoff A, Rand H (2015). CFSAN SNP Pipeline: an automated method for constructing SNP matrices from next-generation sequence data. PeerJ Computer Science.

[5] Sahl JW, Lemmer D, Travis J, Schupp JM, Gillece JD, Aziz M (2016). NASP: an accurate, rapid method for the identification of SNPs in WGS datasets that supports flexible input and output formats. Microb Genom.

[6] Orloski K, Robbe-Austerman S, Stuber T, Hench B, Schoenbaum M (2018). Whole genome sequencing of mycobacterium bovis isolated from livestock in the United States, 1989–2018. Front Vet Sci.

[7] Kamath PL, Foster JT, Drees KP, Luikart G, Quance C, Anderson NJ (2016). Genomics reveals historic and contemporary transmission dynamics of a bacterial disease among wildlife and livestock. Nat Commun.

[8] Hicks J, Stuber T, Lantz K, Erdman M, Robbe-Austerman S, Huang X (2018). Genomic diversity of Taylorella equigenitalis introduced into the United States from 1978 to 2012. PLoS One.

[9] Lorente-Leal V, Farrell D, Romero B, Alvarez J, de Juan L, Gordon SV (2021). Performance and agreement between WGS variant calling pipelines used for bovine tuberculosis control: toward international standardization. Front Vet Sci.

[10] Anaconda. Available from: https://www.anaconda.com/.

[11] Li H. Aligning sequence reads, clone sequences and assembly contigs with BWA-MEM. 2013. arXiv:13033997v1 [q-bioGN]. 10.48550/arXiv.1303.3997.

[12] Li H, Durbin R (2009). Fast and accurate short read alignment with burrows-wheeler transform. Bioinformatics.

[13] Li H, Handsaker B, Wysoker A, Fennell T, Ruan J, Homer N (2009). The sequence alignment/map format and SAMtools. Bioinformatics.

[14] Garrison E, Marth G. Haplotype-based variant detection from short-read sequencing. arXiv. 2012(1207.3907). 10.48550/arXiv.1207.3907.

[15] Hicks J. Available from: https://github.com/jameshicks/vcffilter.

[16] Bankevich A, Nurk S, Antipov D, Gurevich AA, Dvorkin M, Kulikov AS (2012). SPAdes: a new genome assembly algorithm and its applications to single-cell sequencing. J Comput Biol.

[17] Robinson JT, Thorvaldsdottir H, Winckler W, Guttman M, Lander ES, Getz G (2011). Integrative genomics viewer. Nat Biotechnol.

[18] Pozo P, Lorente-Leal V, Robbe-Austerman S, Hicks J, Stuber T, Bezos J (2022). Use of whole-genome sequencing to unravel the genetic diversity of a prevalent mycobacterium bovis spoligotype in a multi-host scenario in Spain. Front Microbiol.

[19] Perera O, Perea C, Davalos E, Flores V, Salazar G, Rosas C (2022). Whole genome sequencing links Mycobacterium bovis from cattle, cheese and humans in Baja California. Recent Adv Bovine Tuber.

[20] Buss BF, Keyser-Metobo A, Rother J, et al. Possible Airborne Person-to-Person Transmission of Mycobacterium bovis — Nebraska 2014–2015. MMWR Morb Mortal Wkly Rep. 2016;65:197–201. 10.15585/mmwr.mm6508a1. 10.15585/mmwr.mm6508a1 26938831

[21] USDA:APHIS:VS:Center for epidemiology and animal health. epidemiologic analyses of virulent newcastle disease in poultry in California, March 2021. USDA-APHIS; 2021. https://www.aphis.usda.gov/animal_health/downloads/animal_diseases/ai/epi-analy-vnd-poultry-calif.pdf.

[22] Glaser L, Carstensen M, Shaw S, Robbe-Austerman S, Wunschmann A, Grear D (2016). Descriptive epidemiology and whole genome sequencing analysis for an outbreak of bovine tuberculosis in beef cattle and white-tailed deer in northwestern Minnesota. PLoS One.

[23] Lakin SM, O’Donnell V, Xu L, Barrette RW, Barnabei J, Nunez R (2022). Whole genome sequencing and molecular epidemiology of the 2021 African swine fever virus outbreak in the Dominican Republic.

[24] Price-Carter M, Brauning R, De Lisle GW, Livingstone P, Neill M, Sinclair J (2018). Whole genome sequencing for determining the source of Mycobacterium bovis infections in livestock herds and wildlife in New Zealand. Front Vet Sci.

[25] Ortiz AP, Perea C, Davalos E, Velázquez EF, González KS, Camacho ER (2021). Whole genome sequencing links mycobacterium bovis from cattle, cheese and humans in Baja California, Mexico. Front Vet Sci.

[26] Quance C, Robbe-Austerman S, Stuber T, Brignole T, DeBess EE, Boyd L (2016). Identification of source of Brucella suis infection in human by whole-genome sequencing, United States and Tonga. Emerg Infect Dis.

[27] Srednik ME, Morningstar-Shaw BR, Hicks JA, Mackie TA, Schlater LK (2022). Antimicrobial resistance and genomic characterization of Salmonella enterica serovar Senftenberg isolates in production animals from the United States. Front Microbiol.

[28] Srednik ME, Lantz K, Hicks JA, Morningstar-Shaw BR, Mackie TA, Schlater LK (2021). Antimicrobial resistance and genomic characterization of Salmonella Dublin isolates in cattle from the United States. PLoS One.

[29] Thacker TC, Palmer MV, Robbe-Austerman S, Stuber TP, Waters WR (2015). Anatomical distribution of Mycobacterium bovis genotypes in experimentally infected white-tailed deer. Vet Microbiol.

[30] Srednik ME, Perea CA, Giacoboni GI, Hicks JA, Foxx CL, Harris B (2023). Genomic features of antimicrobial resistance in staphylococcus pseudintermedius isolated from dogs with pyoderma in Argentina and the United States: A Comparative Study. Int J Mol Sci.

[31] Abadi S, Azouri D, Pupko T, Mayrose I (2019). Model selection may not be a mandatory step for phylogeny reconstruction. Nat Commun.

[32] Li H (2021). New strategies to improve minimap2 alignment accuracy. Bioinformatics.

