# Supplementary material for: vSNP: a SNP pipeline for the generation of transparent SNP matrices and phylogenetic trees from whole genome sequencing data sets
Source: BMC Genomics. 2024 Jun 1;25:545. doi: 10.1186/s12864-024-10437-5 (PMC11143592; doi:10.1186/s12864-024-10437-5)
Supplement: Supplementary file 3 — Supplementary Material 3. [file 12864_2024_10437_MOESM3_ESM.pptx]

## Slide 1
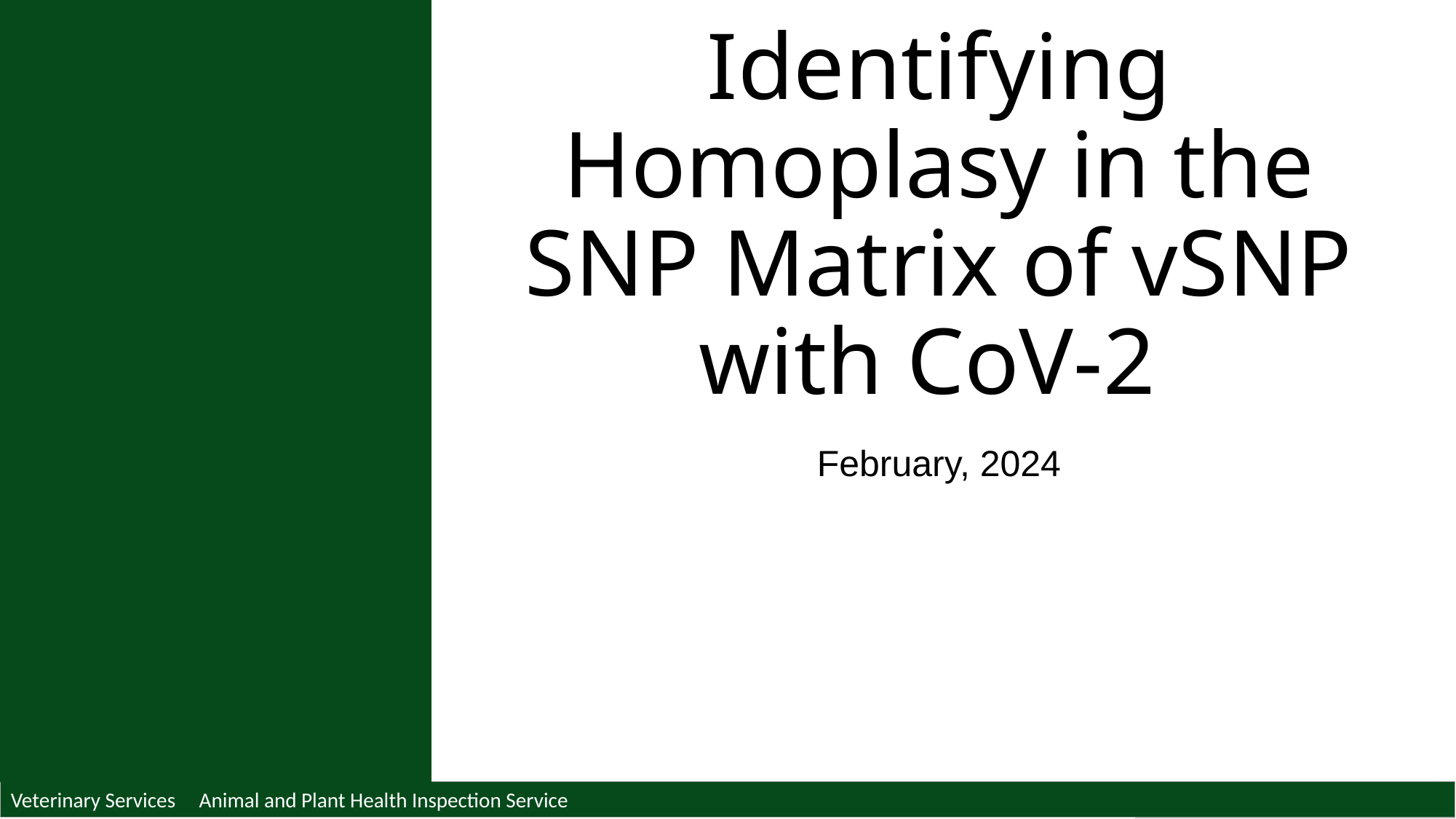

# Identifying Homoplasy in the SNP Matrix of vSNP with CoV-2
February, 2024

## Slide 2
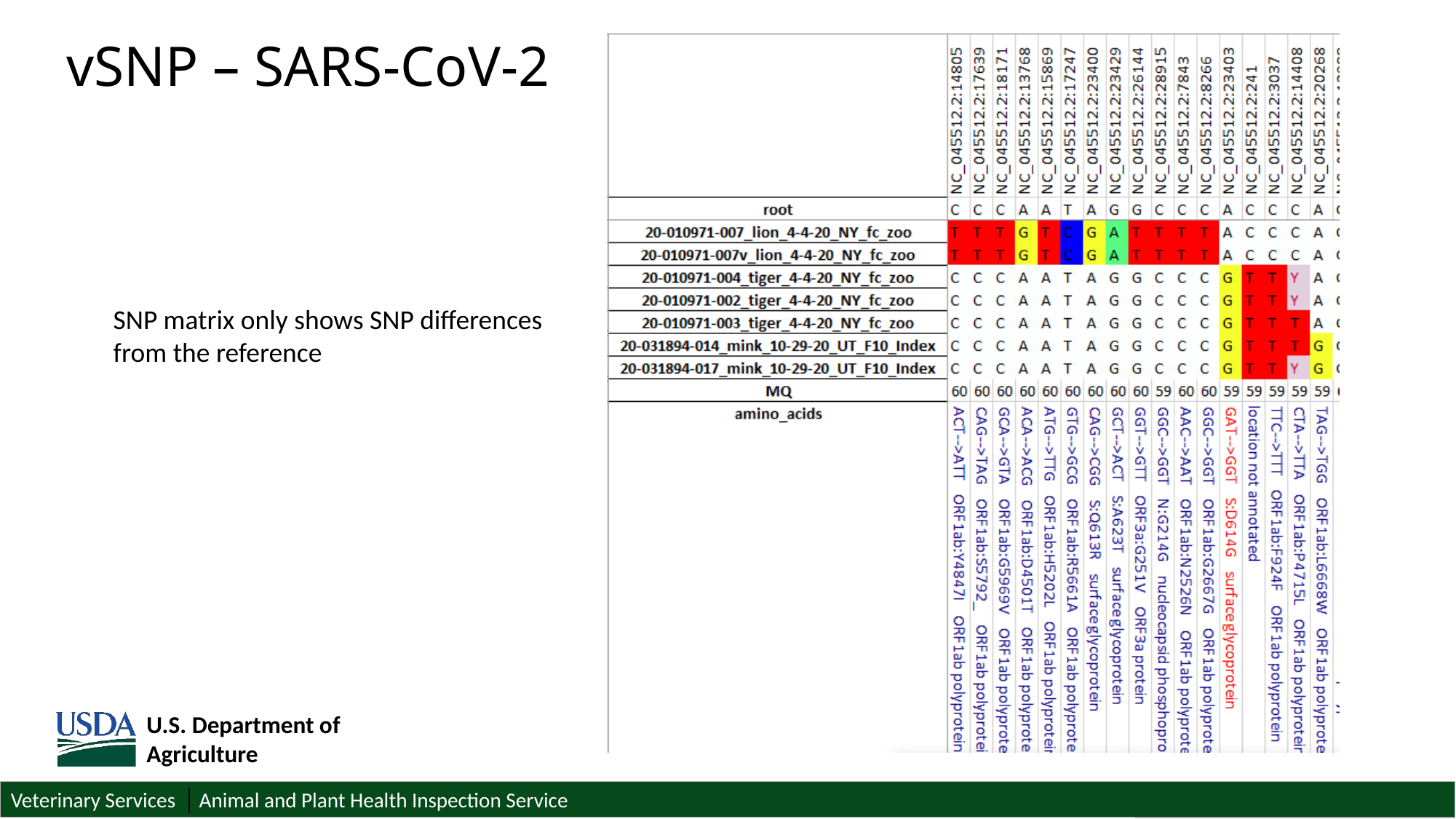

vSNP – SARS-CoV-2
SNP matrix only shows SNP differences
from the reference

## Slide 3
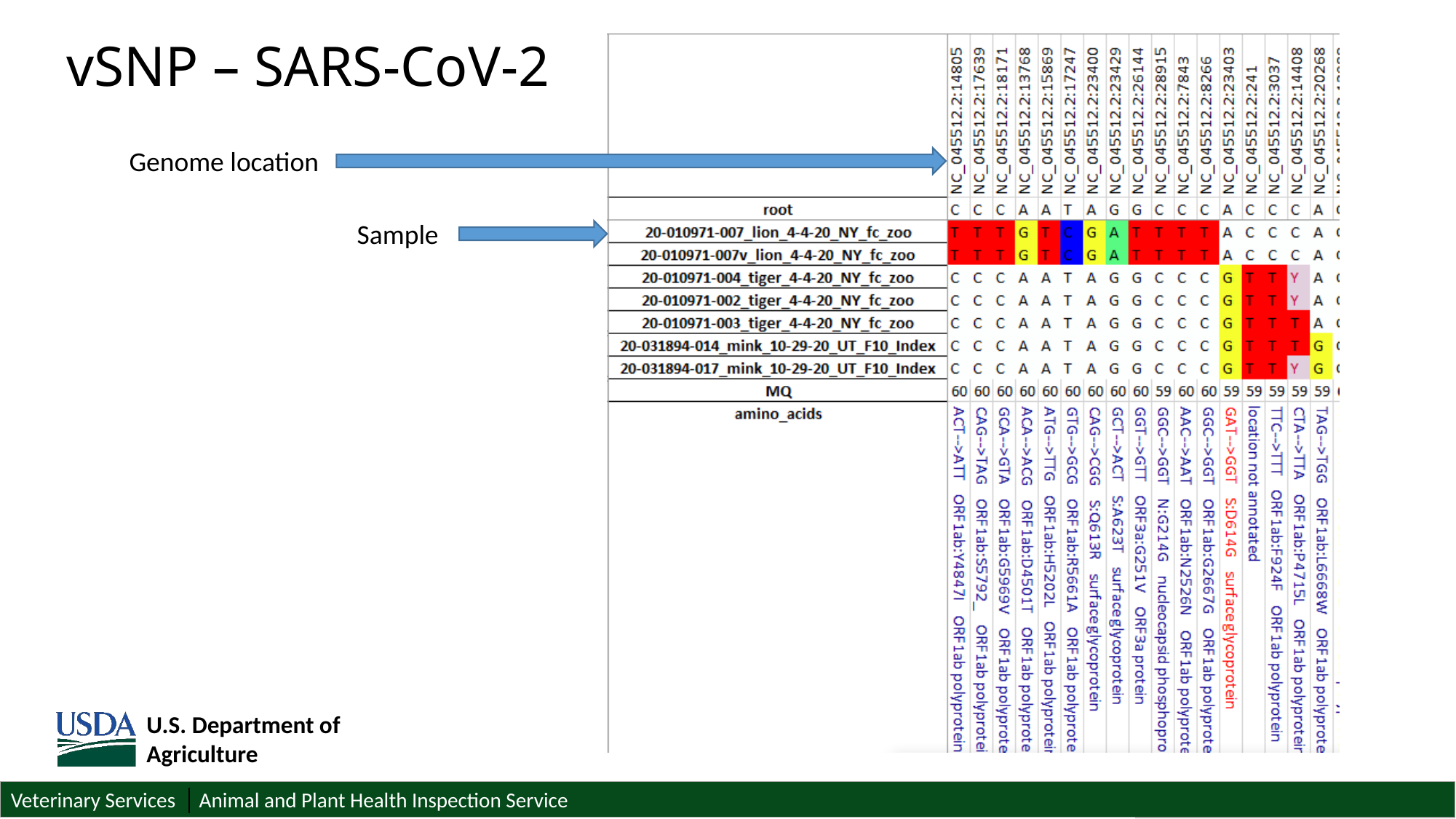

vSNP – SARS-CoV-2
Genome location
Sample

## Slide 4
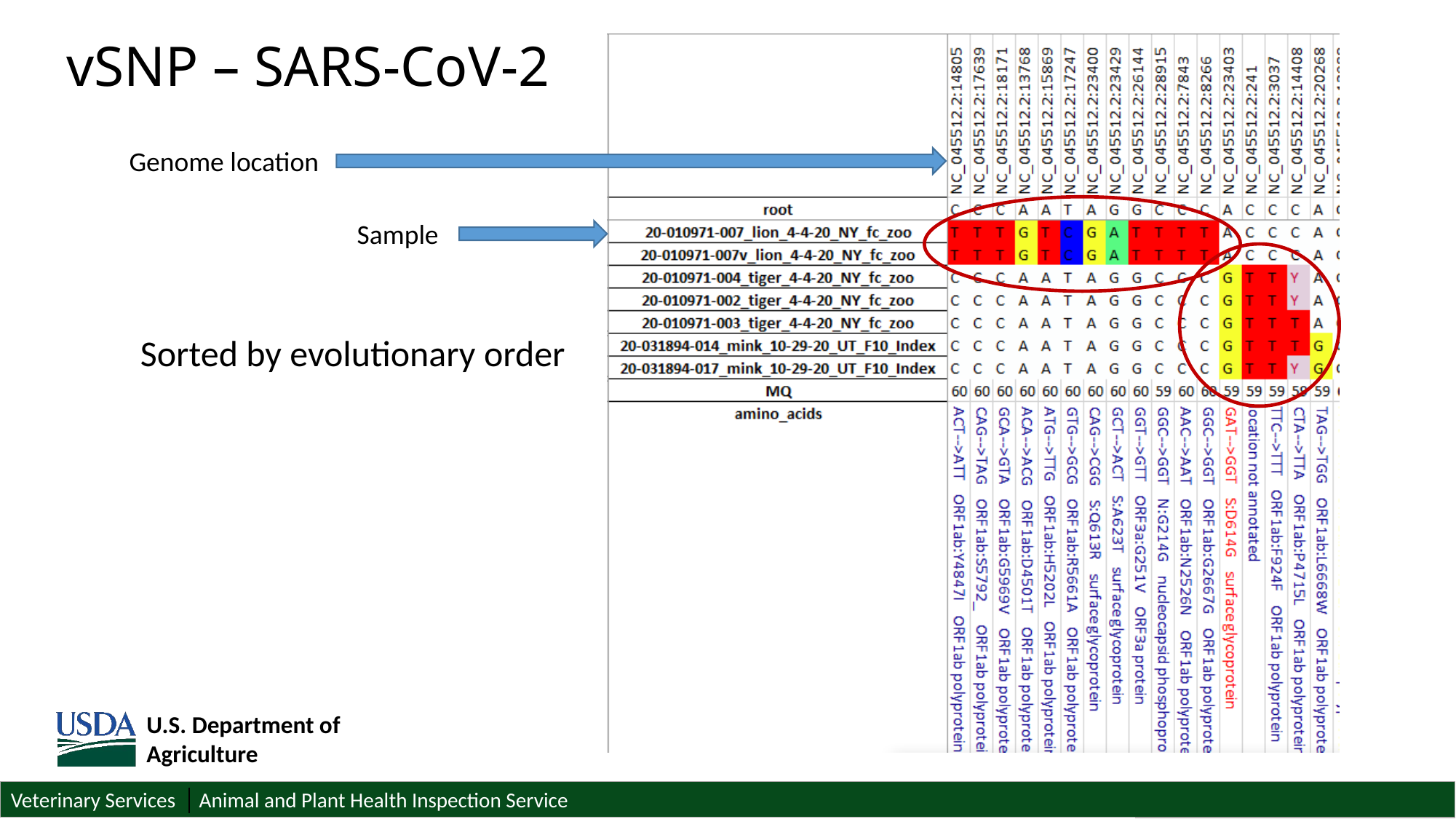

vSNP – SARS-CoV-2
Genome location
Sample
Sorted by evolutionary order

## Slide 5
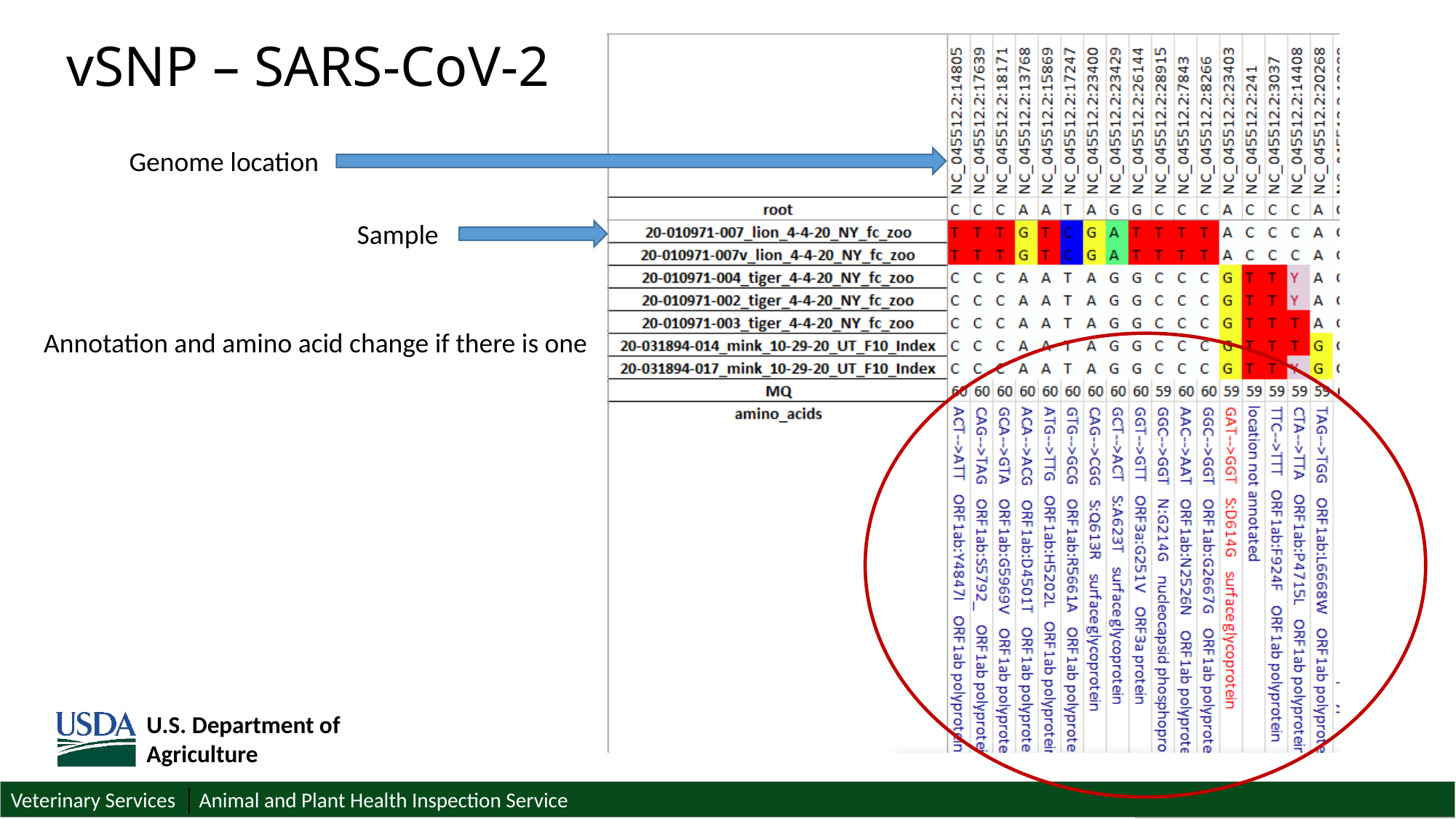

vSNP – SARS-CoV-2
Genome location
Sample
Annotation and amino acid change if there is one

## Slide 6
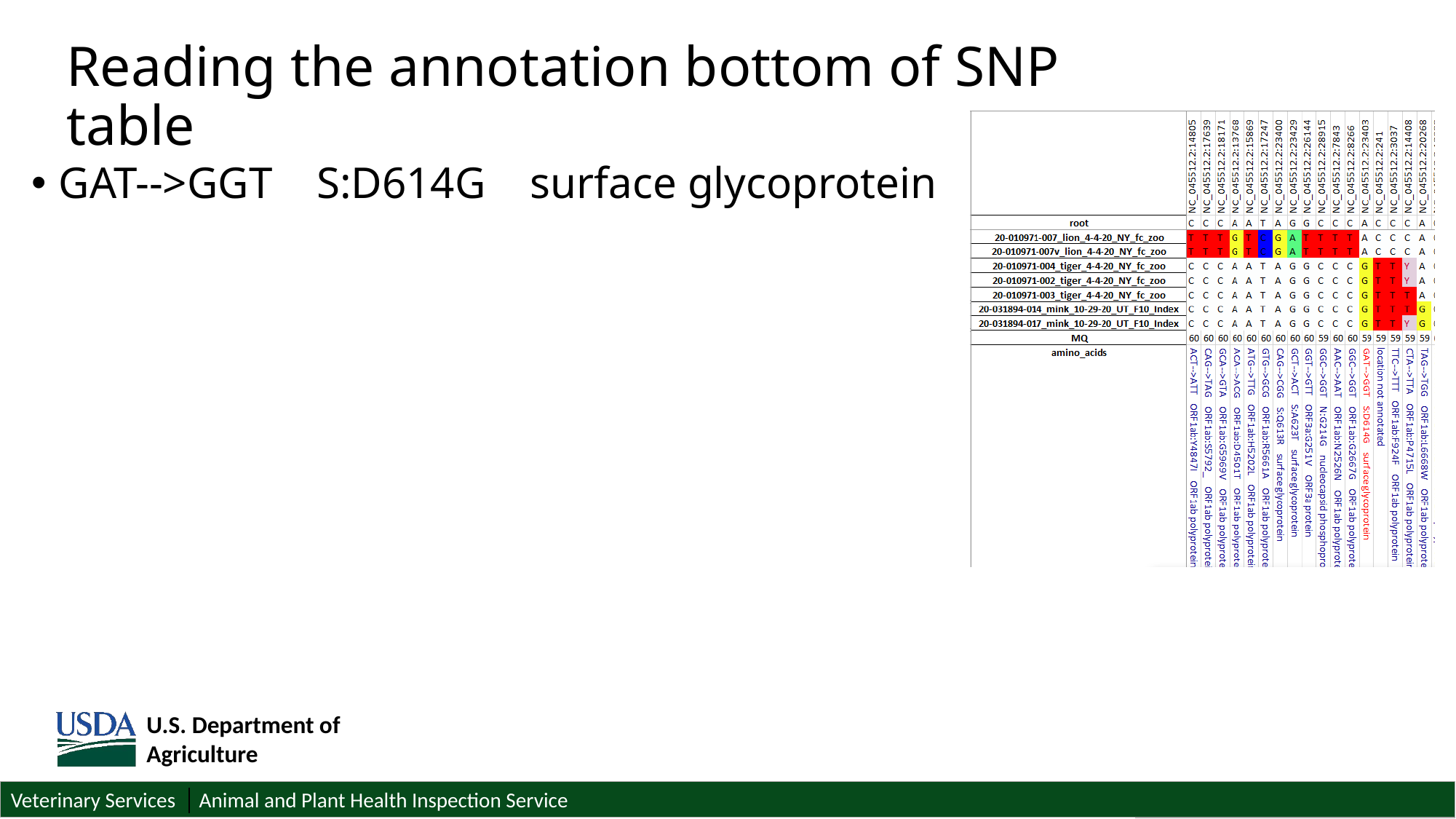

Reading the annotation bottom of SNP table
GAT-->GGT S:D614G surface glycoprotein

## Slide 7
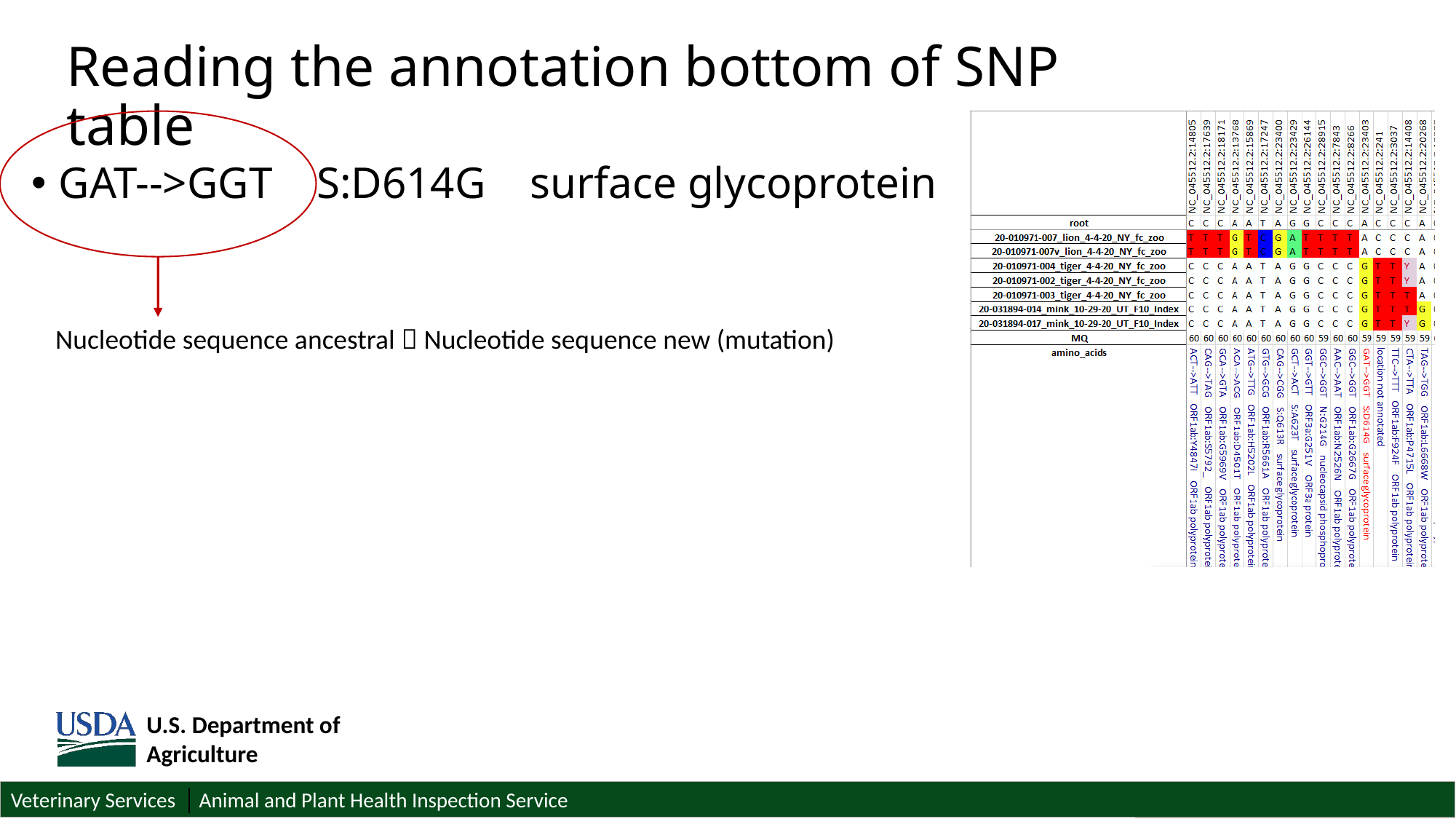

Reading the annotation bottom of SNP table
GAT-->GGT S:D614G surface glycoprotein
Nucleotide sequence ancestral  Nucleotide sequence new (mutation)

## Slide 8
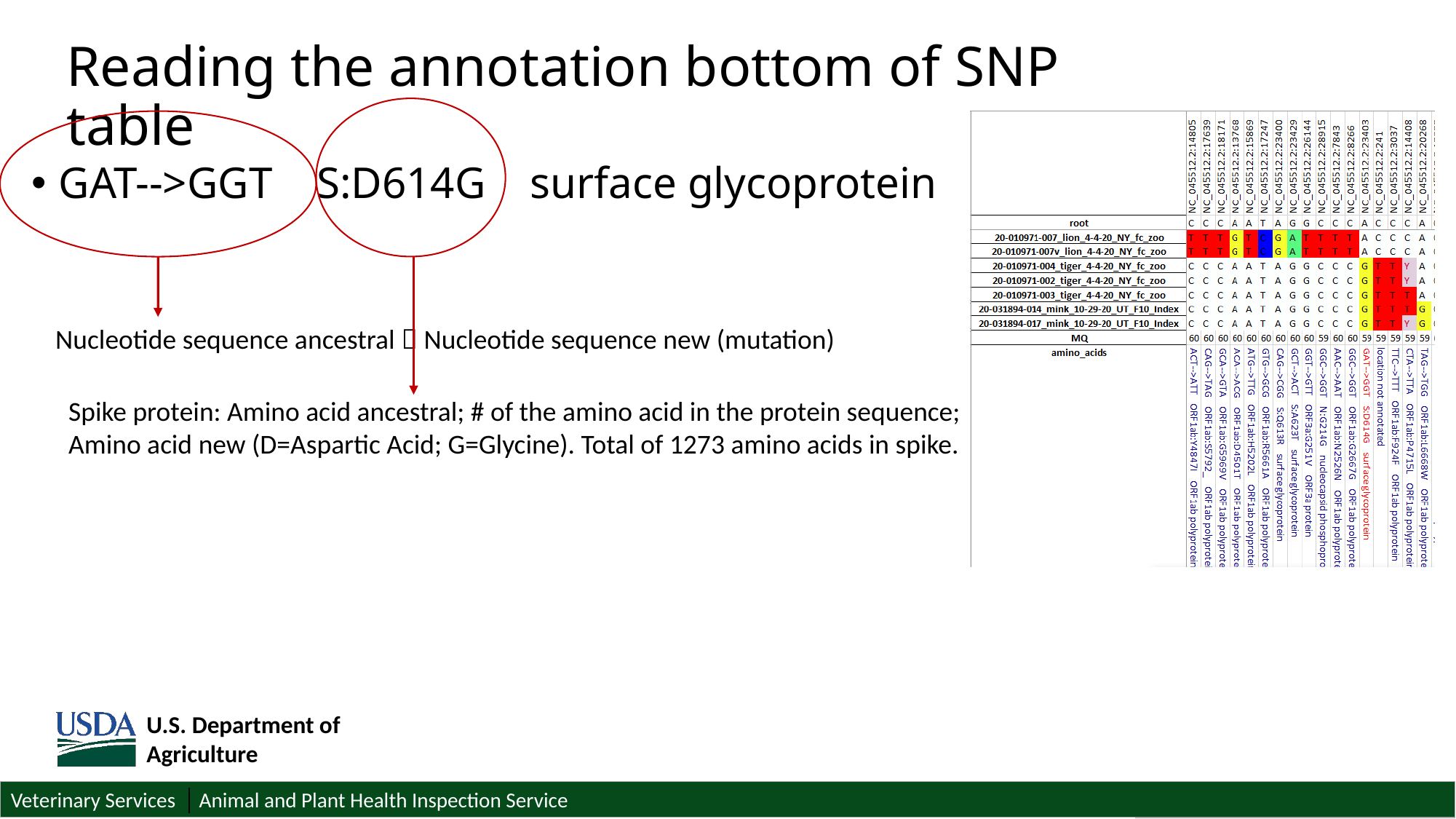

Reading the annotation bottom of SNP table
GAT-->GGT S:D614G surface glycoprotein
Nucleotide sequence ancestral  Nucleotide sequence new (mutation)
Spike protein: Amino acid ancestral; # of the amino acid in the protein sequence;
Amino acid new (D=Aspartic Acid; G=Glycine). Total of 1273 amino acids in spike.

## Slide 9
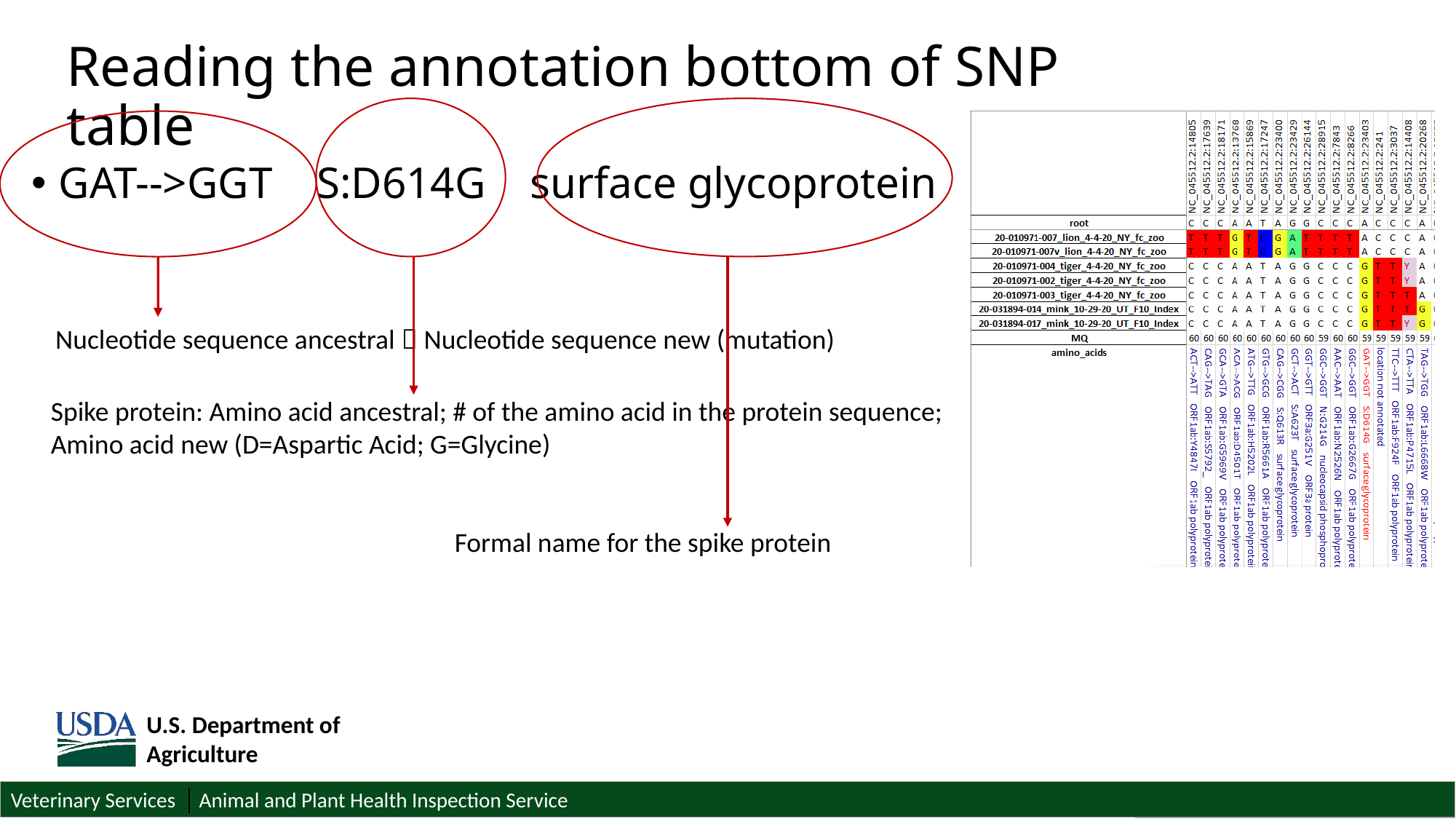

Reading the annotation bottom of SNP table
GAT-->GGT S:D614G surface glycoprotein
Nucleotide sequence ancestral  Nucleotide sequence new (mutation)
Spike protein: Amino acid ancestral; # of the amino acid in the protein sequence;
Amino acid new (D=Aspartic Acid; G=Glycine)
Formal name for the spike protein

## Slide 10
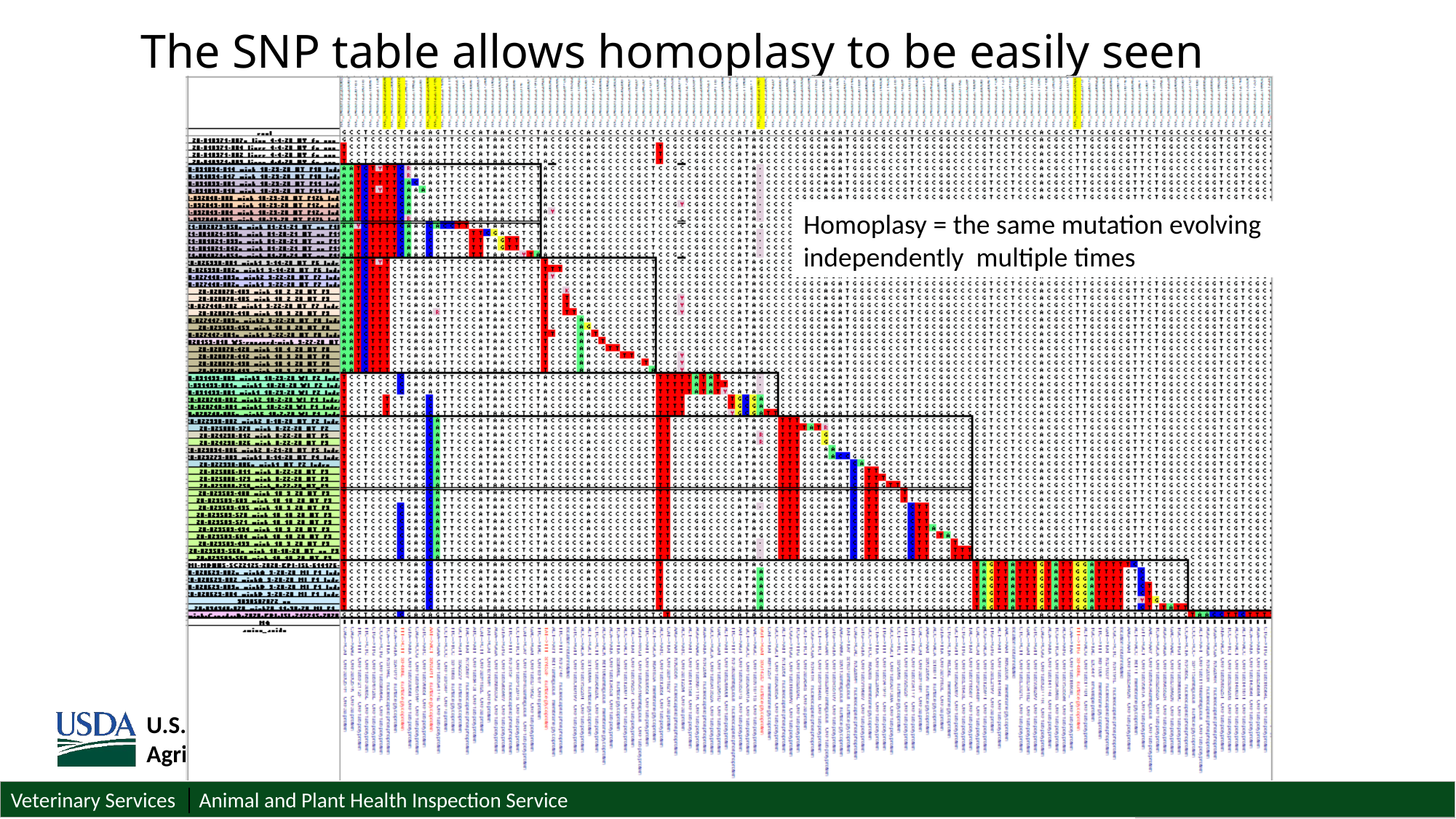

The SNP table allows homoplasy to be easily seen
Homoplasy = the same mutation evolving independently multiple times

## Slide 11
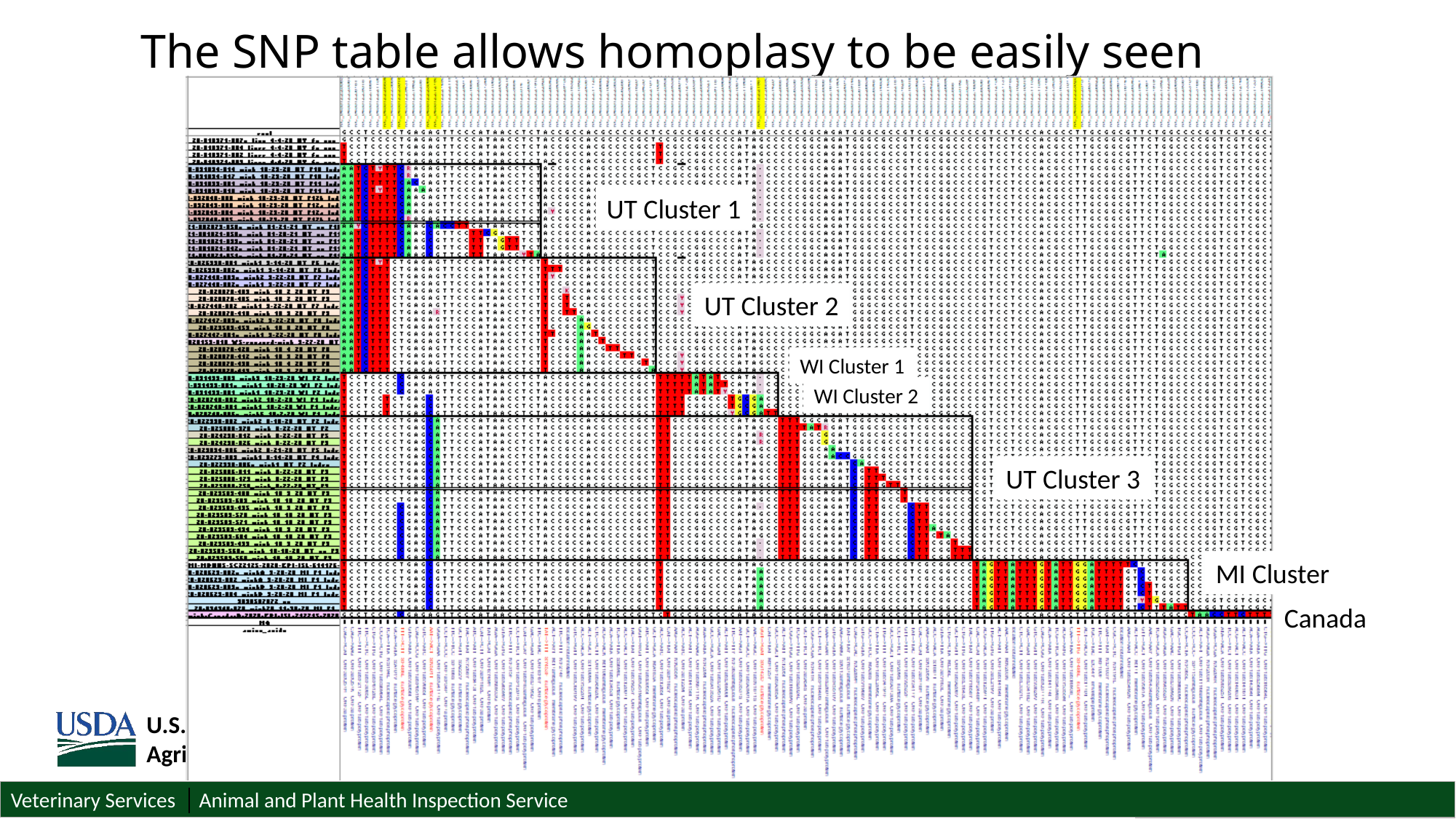

The SNP table allows homoplasy to be easily seen
UT Cluster 1
UT Cluster 2
WI Cluster 1
WI Cluster 2
UT Cluster 3
MI Cluster
Canada

## Slide 12
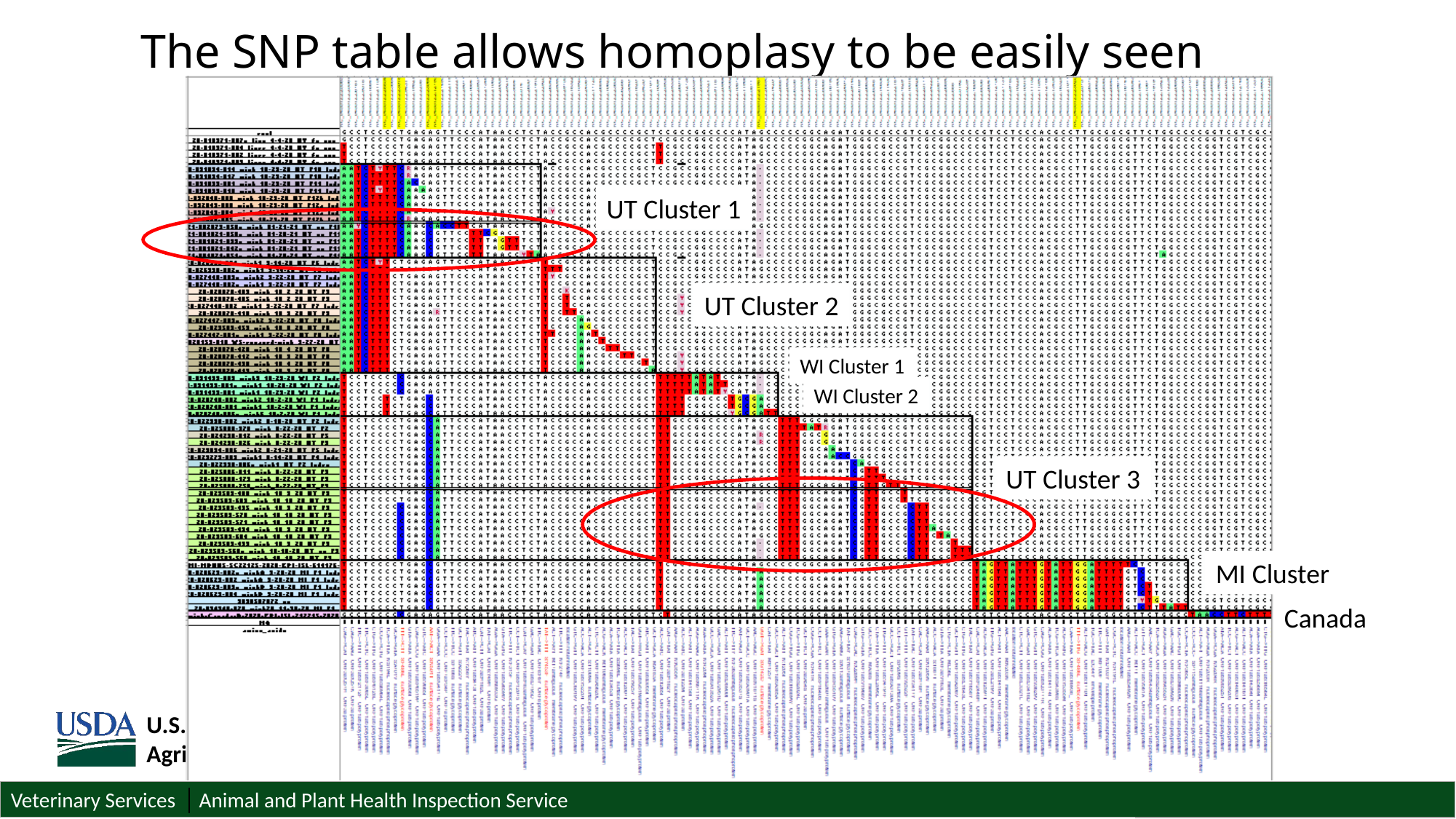

The SNP table allows homoplasy to be easily seen
UT Cluster 1
UT Cluster 2
WI Cluster 1
WI Cluster 2
UT Cluster 3
MI Cluster
Canada

## Slide 13
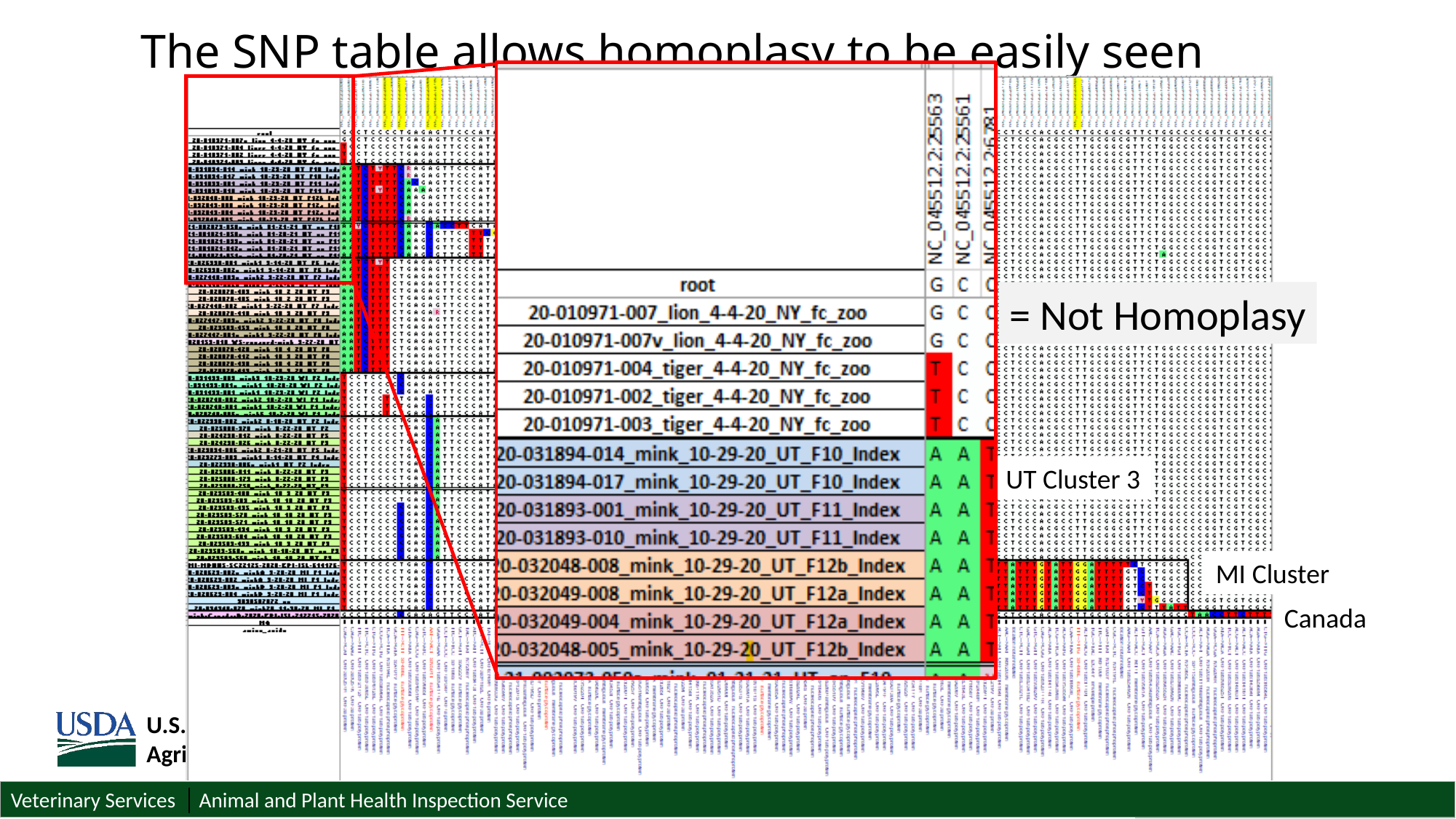

The SNP table allows homoplasy to be easily seen
UT Cluster 1
= Not Homoplasy
UT Cluster 2
WI Cluster 1
WI Cluster 2
UT Cluster 3
MI Cluster
Canada

## Slide 14
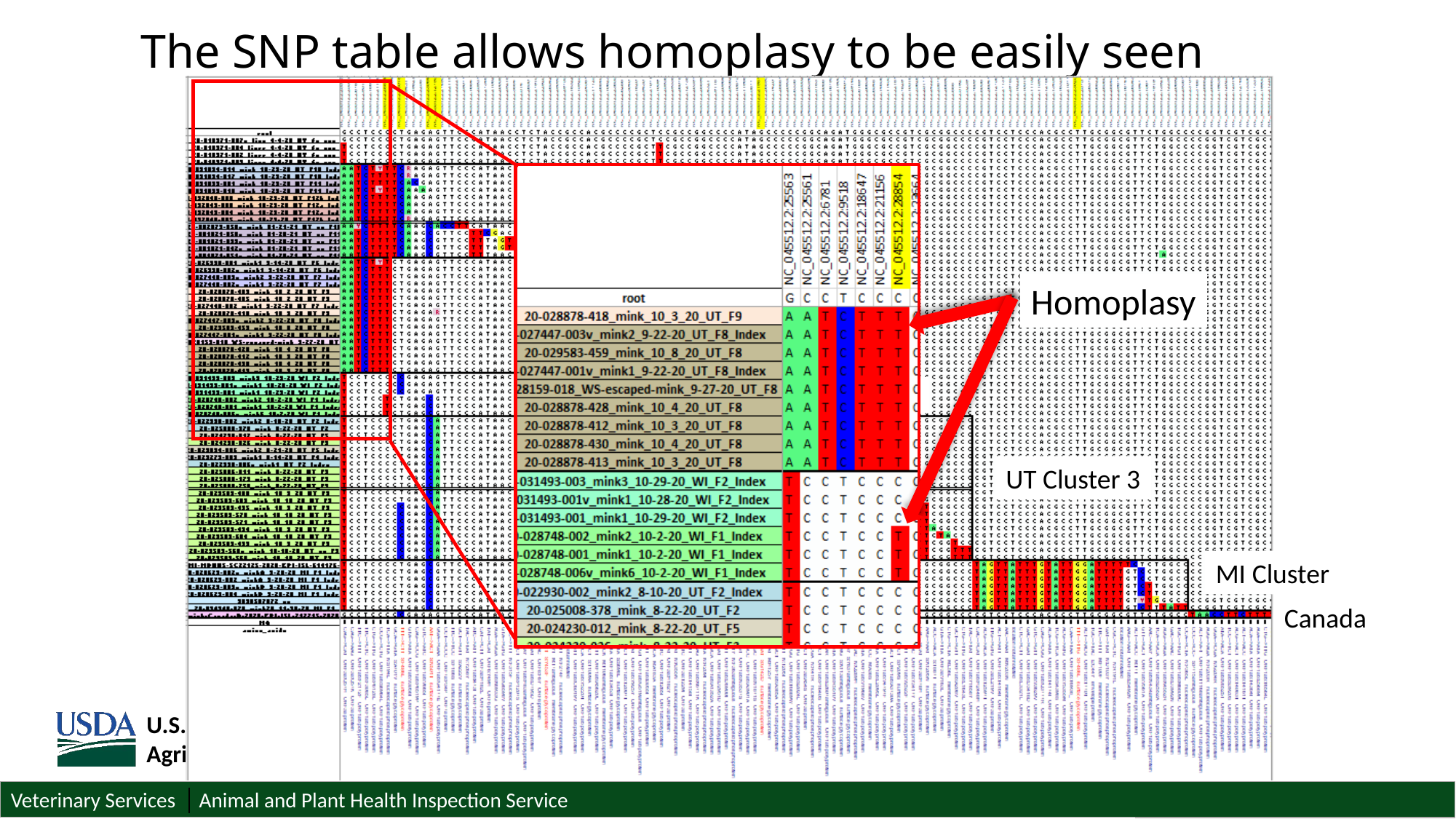

The SNP table allows homoplasy to be easily seen
UT Cluster 1
Homoplasy
UT Cluster 2
WI Cluster 1
WI Cluster 2
UT Cluster 3
MI Cluster
Canada

## Slide 15
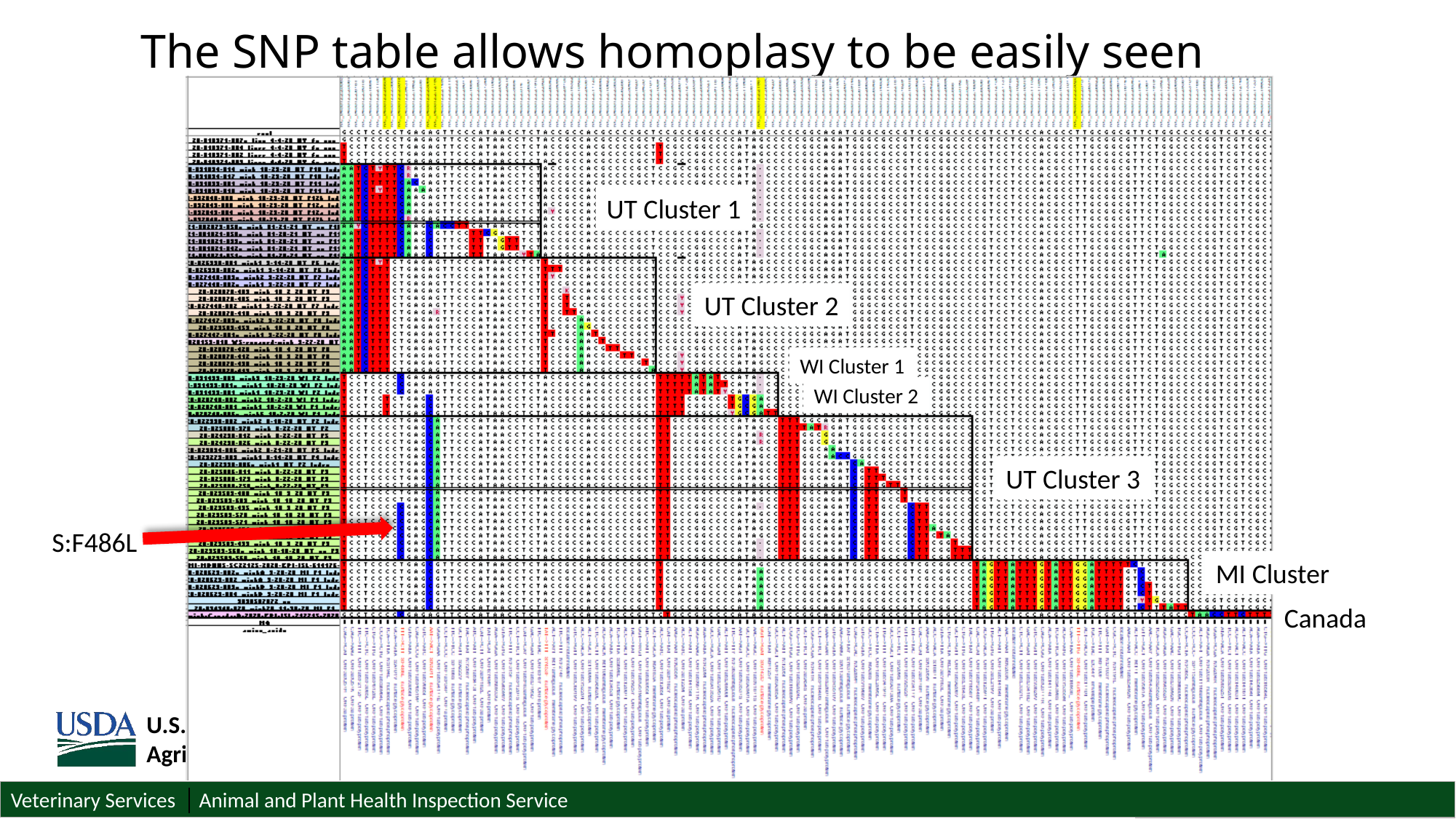

The SNP table allows homoplasy to be easily seen
UT Cluster 1
UT Cluster 2
WI Cluster 1
WI Cluster 2
UT Cluster 3
S:F486L
MI Cluster
Canada

## Slide 16
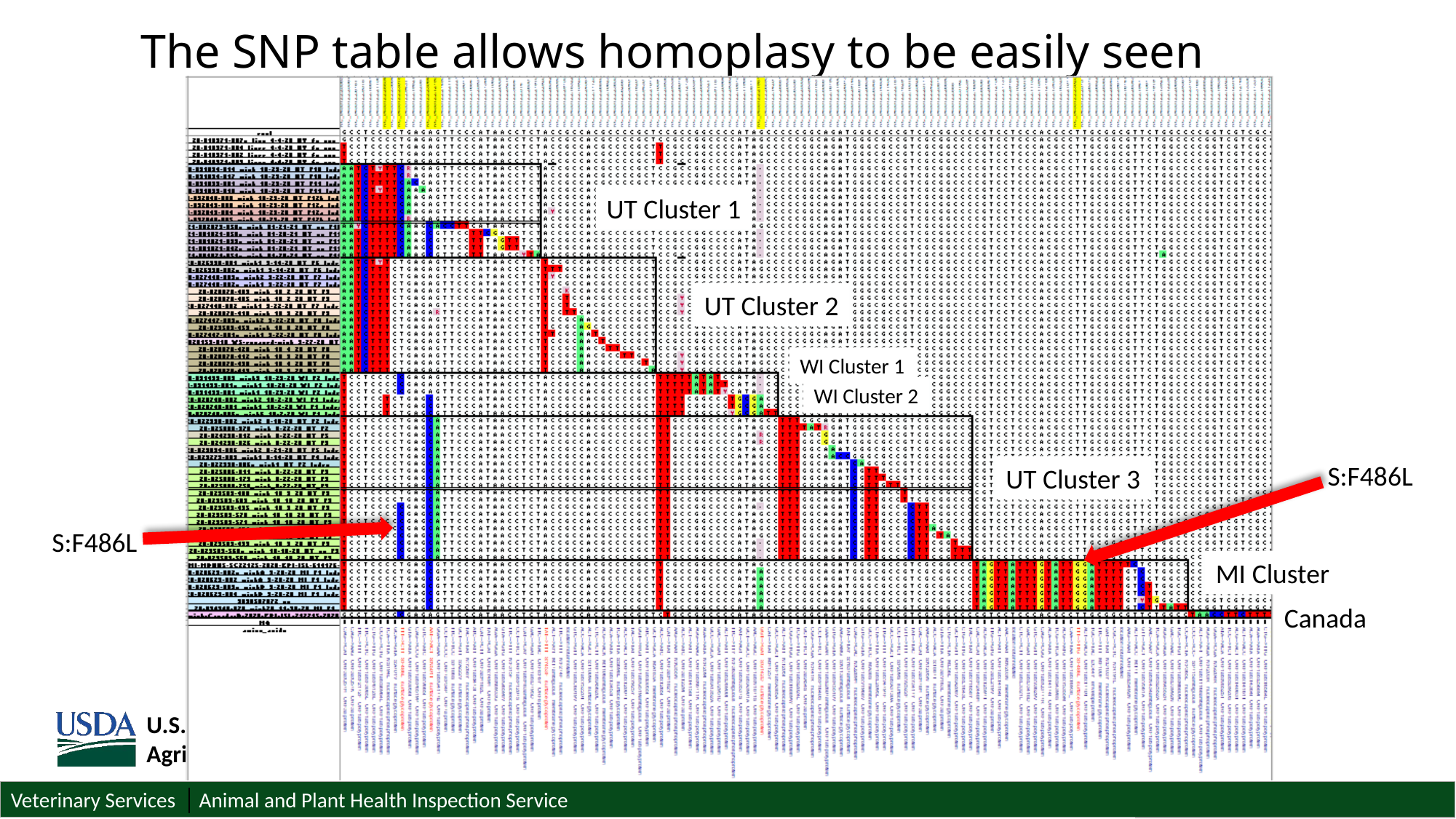

The SNP table allows homoplasy to be easily seen
UT Cluster 1
UT Cluster 2
WI Cluster 1
WI Cluster 2
S:F486L
UT Cluster 3
S:F486L
MI Cluster
Canada

## Slide 17
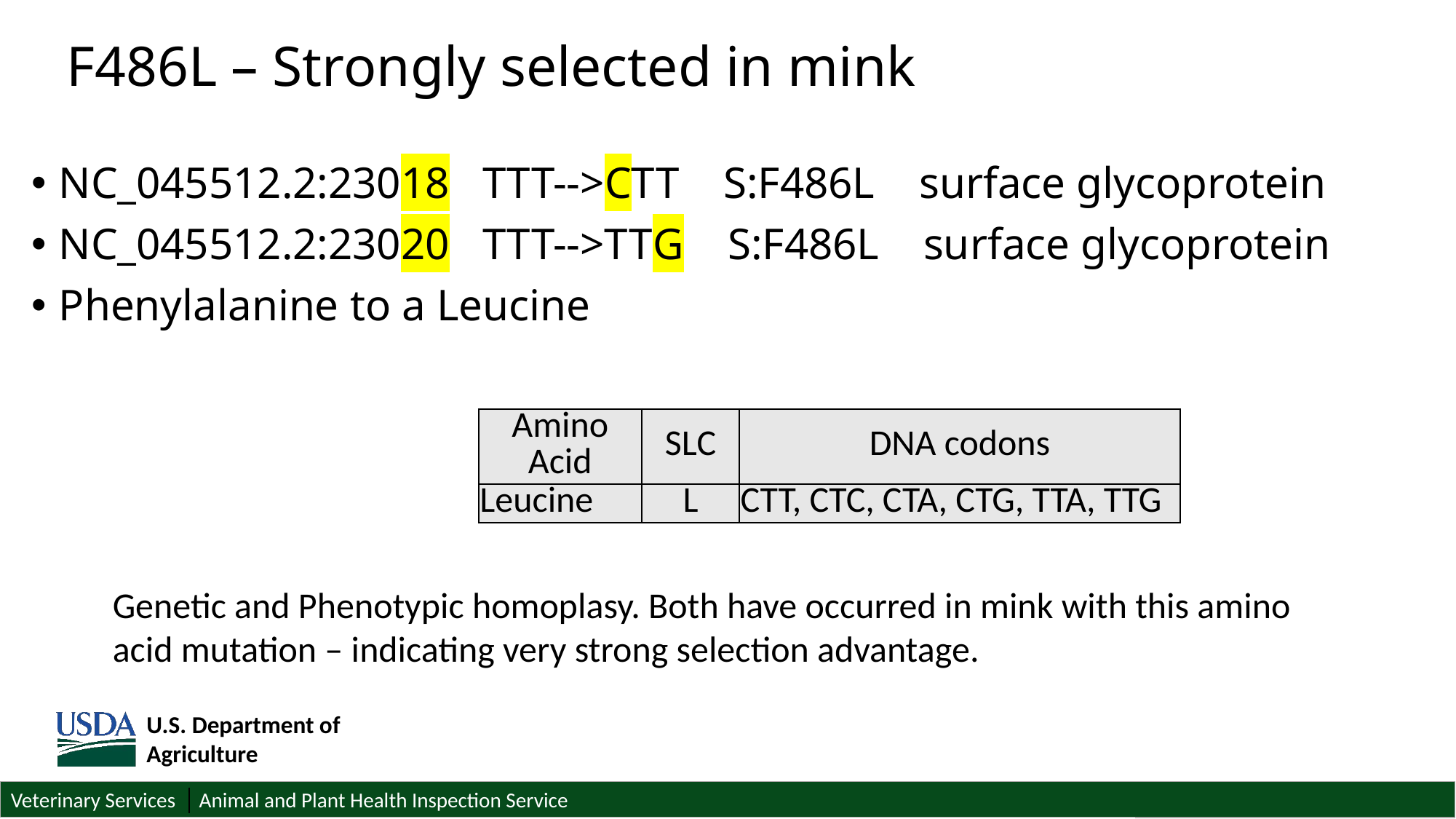

F486L – Strongly selected in mink
NC_045512.2:23018 TTT-->CTT S:F486L surface glycoprotein
NC_045512.2:23020 TTT-->TTG S:F486L surface glycoprotein
Phenylalanine to a Leucine
| Amino Acid | SLC | DNA codons |
| --- | --- | --- |
| Leucine | L | CTT, CTC, CTA, CTG, TTA, TTG |
Genetic and Phenotypic homoplasy. Both have occurred in mink with this amino acid mutation – indicating very strong selection advantage.
