# Supplementary material for: vSNP: a SNP pipeline for the generation of transparent SNP matrices and phylogenetic trees from whole genome sequencing data sets
Source: BMC Genomics. 2024 Jun 1;25:545. doi: 10.1186/s12864-024-10437-5 (PMC11143592; doi:10.1186/s12864-024-10437-5)

**Phylogenetic tree consistency under different models of evolution**

*Taylorella equigenitalis* phylogenetic tree (from Figure 2) generated under different models of evolution.

**JC69**


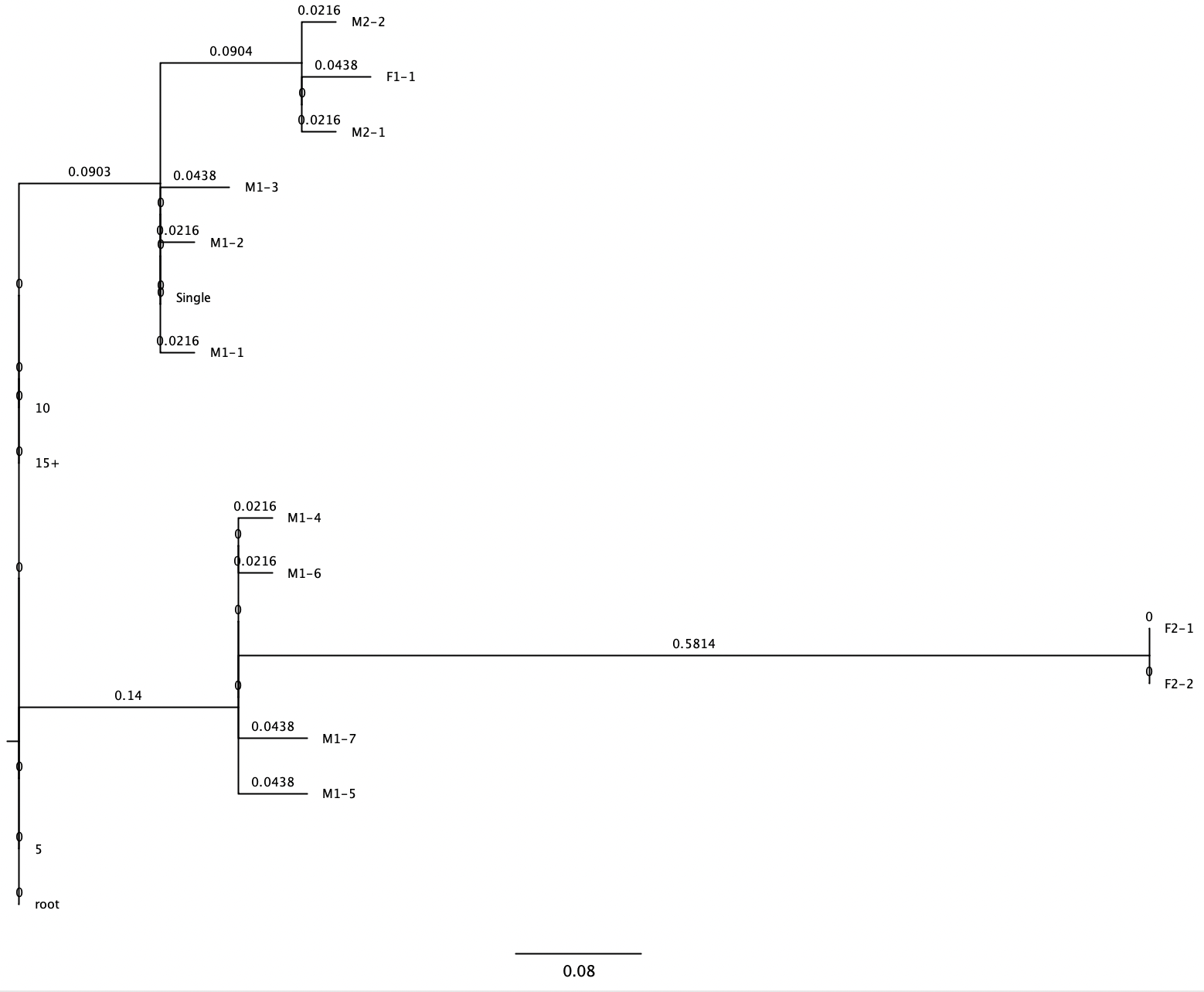


**GTR-GAMMA**


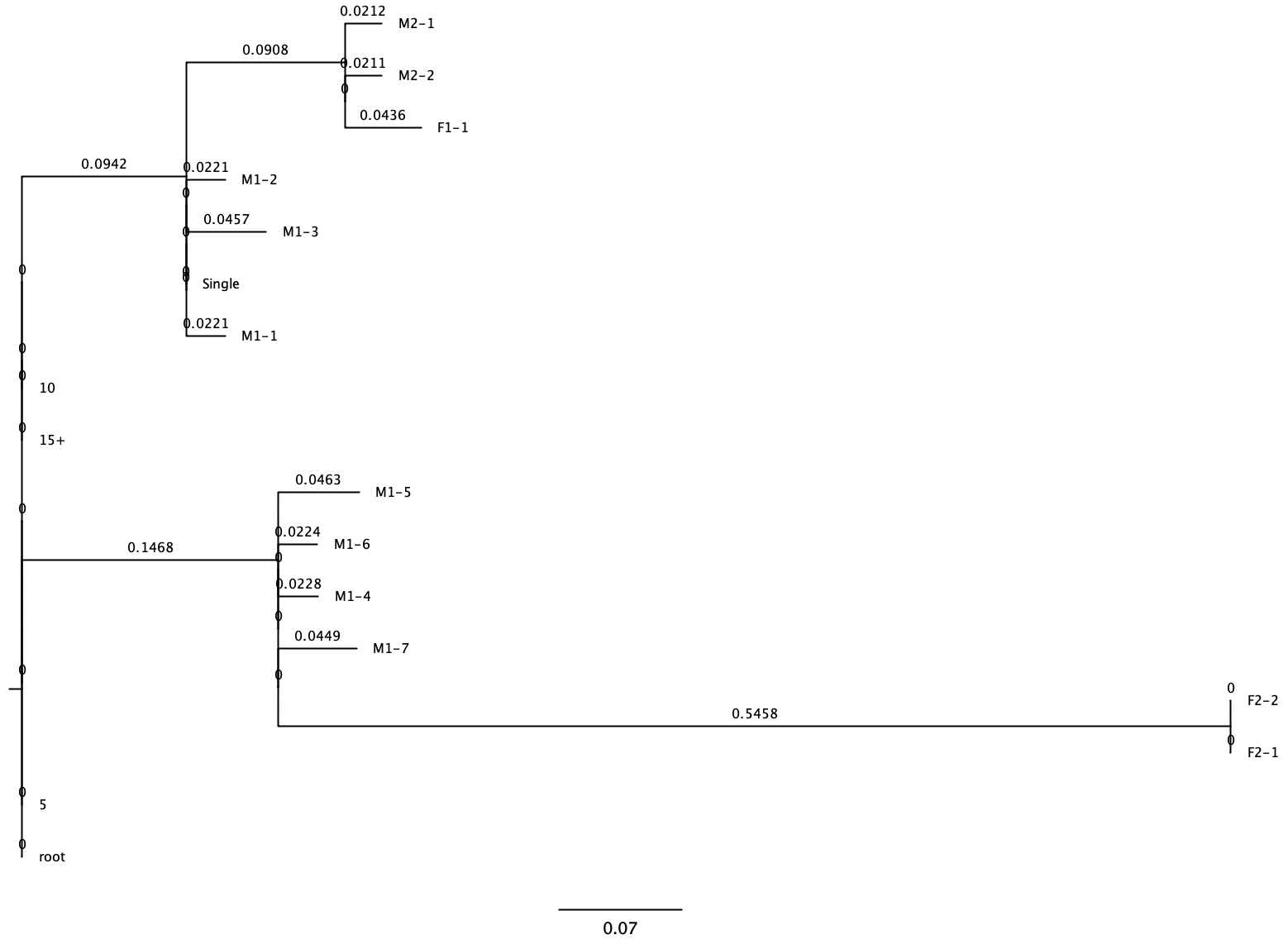


**GTR-CATI**


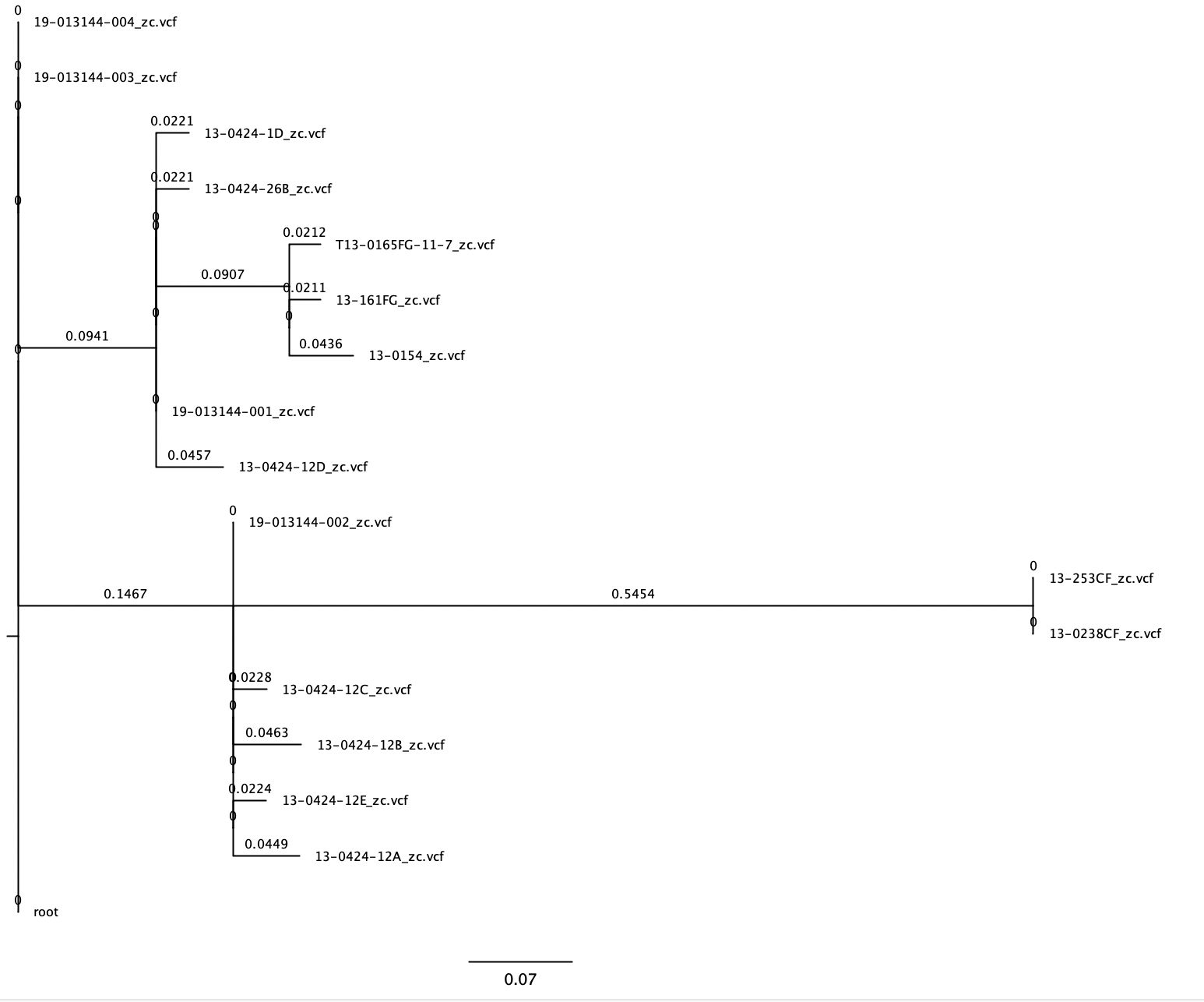

Supplement: Supplementary file 4 — Supplementary Material 4. [file 12864_2024_10437_MOESM4_ESM.docx]
